# Choose Your Level Wisely: Assessing Density Functionals and Dispersion Corrections for Metal Carbonyl Compounds

**DOI:** 10.1002/jcc.70245

**Published:** 2025-10-10

**Authors:** Vinícius Glitz, Vinícius Capriles Port, Ebbe Nordlander, Rosely Aparecida Peralta, Giovanni Finoto Caramori

**Affiliations:** ^1^ Departamento de Química Universidade Federal de Santa Catarina Florianópolis Brazil; ^2^ Chemical Physics, Department of Chemistry Lund University Lund Sweden

**Keywords:** dispersion corrections, DLPNO‐CCSD(T), KS‐DFT, manganese(I), rhenium(I)

## Abstract

Understanding the structure of metal‐ligand complexes is essential for catalyst design, materials development, and biochemical modeling. Metal carbonyls are especially relevant due to their diverse structures and electronic features. Here, we benchmarked seventeen density functionals (B3LYP, BP86, CAM–B3LYP, M06, M06L, PBE, PBE0, r^2^SCAN, r^2^SCAN–3c, revPBE, revTPSS, RPBE, TPSS, TPSS0, TPSSh, ωB97, and ωB97X) combined with three dispersion schemes (D3zero, D3BJ, D4) and also tested calculations without dispersion correction, totaling fifty‐four approaches. Their ability to reproduce geometries, structural parameters, and CO stretching frequencies was assessed for thirty‐four Mn(I) and Re(I) carbonyls obtained from the CCDC. Relative electronic energies were further compared using DLPNO‐CCSD(T) calculations, alongside evaluation of computational cost. Our results highlight that hybrid meta‐GGA and meta‐GGA functionals, particularly TPSSh(D3zero) and r^2^SCAN(D3BJ, D4), offer the best balance between accuracy and efficiency, providing reliable structures, vibration properties, and energetics consistent with high‐level DLPNO‐CCSD(T) references.

## Graphical abstract

Evaluation of 54 functional/dispersion approaches for Mn(I) and Re(I) carbonyls shows that meta‐GGA and hybrid meta‐GGA functionals, particularly TPSSh(D3zero) and r^2^SCAN(D3BJ/D4), achieve the best balance of accuracy and efficiency, reproducing geometries, CO stretching frequencies, and energetics consistent with DLPNO‐CCSD(T).

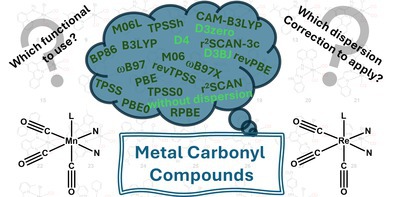

## 1 Introduction

Metal carbonyl compounds have a wide range of applications across various fields of chemistry. This class of compounds finds application in catalytic reactions, including the reduction of carbon dioxide to methanol, carbon monoxide, and formic acid [1, 2, 3, 4].

Other examples of catalysis include hydrogenation reactions, where, e.g., manganese(I) complexes are utilized for the hydrogenation of substrates such as ketones, nitriles, carbon dioxide, and alkynes, as well as for the hydrofunctionalization of unsaturated carbon–carbon bonds, including the dehydrogenative silylation of alkenes and *trans*‐1,2‐diboration of terminal alkynes [5]. The formation of carbon–carbon bonds is also of significance in this context. This is illustrated by allylation reactions between unactivated alkyl iodides and allyl sulfones, which are mediated by visible light [6], and the α‐alkylation of ketones and nitriles using manganese(I) pincer‐NHC complexes [7].

Si‐H and Ge‐H bond activations have been achieved through visible‐light photocatalysis, enabling the hydrosilylation and hydrogermylation of alkynes [8]. Radical‐based processes exemplify the versatility of manganese‐based carbonyl complex catalysts, encompassing atom transfer radical cyclization of unactivated alkyl iodides [9] and the formation of ketyl radicals via atom transfer catalysis [10].

Carbonyl compounds also have biological applications, particularly in the controlled release of carbon monoxide (CO), a molecule recognized for its signaling properties, acting as a vasodilator, neurotransmitter, and anticancer agent [11, 12]. CO release can be triggered enzymatically [13], by pH changes [14], magnetic heating [15], or light irradiation [16, 17].

It is evident from a thorough examination of the relevant literature that computational chemistry plays a crucial role in understanding the applications of this class of compounds [18, 19, 20, 21, 22, 23, 24]. Therefore, it is imperative to employ computational protocols that are sufficiently precise and economical in terms of computational effort to accurately reproduce the structural characteristics and the bonding situations of Mn(I) and Re(I) carbonyl systems. This will ensure the attainment of reliable results. It is also essential that a given computational protocol allows the identification of intermediates and transition states, thereby deepening and broadening the understanding of the mechanisms underlying these applications. This, in turn, supports the rational modification of metal carbonyl complexes to improve their catalytic performance and broaden their scope of application.

In this sense, the Kohn‐Sham density functional theory emerges as a promising option that may fulfill the computational requirements. Nevertheless, given that exchange‐correlation functionals employ a variety of approaches and some are plagued by issues such as self‐interaction error [25], delocalization error [26], inadequate representation of density [27], and unsatisfactory performance for specific systems, it is crucial to evaluate the performance of such functionals to select the most effective ones capable of accurately describing the chemical issues involving this class of compounds.

**FIGURE 1:**
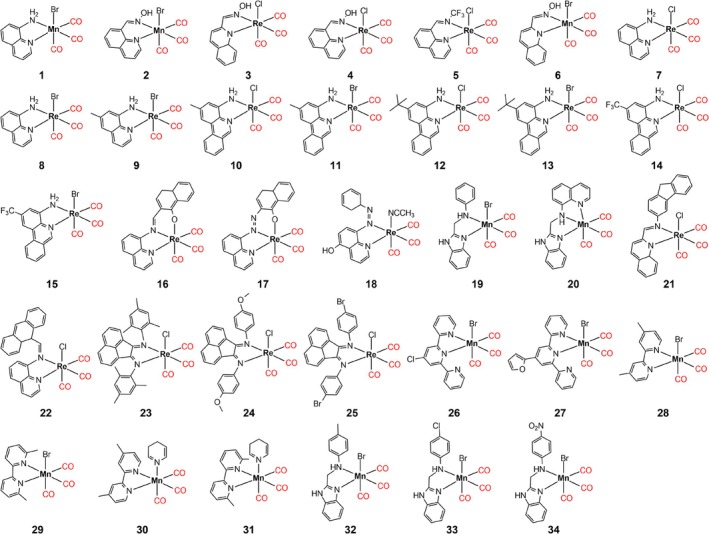
2D representation of Re(I) and Mn(I) compounds used in this study [28, 29, 30, 31, 32, 33, 34, 35, 36, 37, 38, 39].

Consequently, the present study examined thirty‐four coordination compounds, of which twenty contain rhenium(I) and fourteen contain manganese(I) (Figure 1), employing various density functionals and dispersion corrections (Table 1) to assess the computational outcomes through comparison with crystallographic data and carbonyl stretching in the infrared. Rhenium(I) and manganese(I) carbonyl compounds are utilized in numerous chemical processes; therefore, it is imperative to evaluate computational protocols that can accurately and reliably capture their structural and spectroscopic properties. In addition to geometrical and vibration benchmarks, the study also evaluates the optimized structures by computing DLPNO‐CCSD(T) single‐point energies, taken as a high‐level reference for the energetic minimum. Computational cost and time efficiency are further analyzed, allowing for a balanced evaluation between accuracy and practicality. The present benchmark protocol does not restrict the scope of the study; rather, it facilitates the effective investigation of the properties of this class of carbonyl compounds.

**TABLE 1:** Combination of functional and dispersion used in this work.

Functional	D4	D3BJ	D3zero	Without
B3LYP	✓	✓	✓	✓
BP86	✓	✓	✓	✓
CAM–B3LYP	✓	✓	✓	✓
M06	×	×	✓	✓
M06L	×	×	✓	✓
PBE	✓	✓	✓	✓
PBE0	✓	✓	✓	✓
r^2^SCAN	✓	✓	×	✓
r^2^SCAN–3c	×	×	×	✓ ^a^
revPBE	✓	✓	✓	✓
revTPSS	✓	×	×	✓
RPBE	✓	✓	✓	✓
TPSS	✓	✓	✓	✓
TPSS0	✓	✓	✓	✓
TPSSh	✓	✓	✓	✓
ωB97	✓	×	×	✓
ωB97X	✓	×	×	✓

^a^ r^2^SCAN–3c is a composite method based on the r^2^SCAN functional, combined with D4 dispersion and additional corrections.

## 2 Computational Methods

### 2.1 Composition of the Benchmark Set

Initially, thirty‐four structures (Figure 1) were selected from the Cambridge Crystallographic Data Center (CCDC) to ensure high‐quality structural data. The search was based on octahedral coordination compounds with three carbonyl ligands coordinated to the metal center in a facial mode, where we identified compounds with manganese(I) and rhenium(I). The three remaining coordination sites of thirty‐one compounds are occupied by a bidentate nitrogen ligand (of various types) and a monodentate ligand (bromide, chloride, pyridine, or acetonitrile). The other three compounds (**16**, **17**, and **20**) complete the coordination sphere with tridentate ligands. The structures selected are free from disorder and have a low R‐value (with only three structures exceeding 5%; namely 6.8%, 10.52%, and 13.64% for **22**, **21**, and **30**, respectively), indicating high‐quality crystallographic data.

### 2.2 Calculations

Density Functional Theory (DFT) calculations were conducted using the Orca 5.0.4 package [40, 41]. A range of exchange‐correlation functionals, covering different levels of complexity on Perdew's Jacob's ladder, was employed, as summarized in Table 1. The functionals in this selection cover a variety of approaches within DFT, each designed to address specific features in electronic structure calculations.

For instance, BP86 is a GGA functional combining Becke's 1988 exchange with Perdew's 1986 correlation [42, 43]. M06L, a Minnesota family functional, is a meta‐GGA functional optimized for accurately describing non‐local electron correlation effects [44]. M06, another member of the same family, blends hybrid and meta‐GGA characteristics, emphasizing a balanced treatment of exchange and correlation effects [45]. These functionals have been used for the prediction of electronic excitation energies of main group compounds.

B3LYP is a widely used hybrid functional, designed primarily for ground‐state properties, that incorporates a mixture of Becke's exchange and Lee‐Yang‐Parr correlation [46]. This hybrid functional has some drawbacks, as it tends to under‐ or overestimate excitation energies, which can lead to significant discrepancies with experimental data. CAM–B3LYP, ωB97, and ωB97X were developed as alternatives to B3LYP, seeking to incorporate necessary corrections to overcome its limitations, especially those related to the description of excited states.

CAM–B3LYP, proposed by Handy, is a range‐separated hybrid functional, designed to improve the asymptotic behavior of the effective potential in DFT calculations [47]. ωB97 and ωB97X, both devised by Head‐Gordon, belong to the range‐separated hybrid category, with ωB97 offering fully variable Fock exchange and ω97X minimizing Fock exchange to enhance accuracy in electronic structure predictions [48].

PBE is a GGA functional developed by Perdew, Burke, and Ernzerhof, designed to satisfy known exact constraints of the exchange‐correlation energy while avoiding empirical fitting [49]. It is widely applied in solid‐state physics and molecular chemistry due to its balanced performance for structural and energetic properties. PBE0 is a hybrid functional derived from PBE, incorporating a fixed fraction (25%) of exact Hartree‐Fock exchange into the GGA framework [50]. This modification improves thermochemical accuracy and often yields better results for molecular properties compared to pure GGA functionals. revPBE is a revised form of PBE with modifications to the exchange term, aimed at improving the description of binding energies and weak interactions [51]. Although it performs well for certain non‐covalent interactions, it can overestimate equilibrium distances in some cases. RPBE is a GGA functional specifically modified from PBE to improve the accuracy of adsorption energy predictions for molecules on metal surfaces [52]. This makes it particularly relevant in surface science and catalysis studies.

TPSS is a non‐empirical meta‐GGA functional developed by Tao, Perdew, Staroverov, and Scuseria [53]. It was designed to satisfy exact constraints and to provide a more accurate treatment of both molecular and solid‐state systems compared to GGAs. TPSS0 is the hybrid variant of TPSS, incorporating a fixed fraction of Hartree‐Fock exchange thermochemical and spectroscopic predictions [54]. TPSSh is a semi‐hybrid functional based on TPSS, containing a smaller fraction of exact exchange (10%) than TPSS0 [55]. This balance aims to enhance accuracy for transition metal complexes and systems with a delicate interplay between exchange and correlation effects. revTPSS is a revised version of the Tao‐Perdew‐Staroverov‐Scuseria (TPSS) meta‐GGA functional [56]. The revision improves accuracy for lattice constant and cohesive energies in solids, while maintaining good performance for molecular systems.

r^2^SCAN is a regularized version of the strongly constrained and appropriately normed (SCAN) meta‐GGA functional [57]. The regularization improves numerical stability without compromising the accuracy of the original SCAN, making it more robust for a wider range of systems. r^2^SCAN–3c is a composite method based on r^2^SCAN, combined with atom‐pairwise dispersion correction (D4), geometric counterpoise corrections (gCP), and a minimal triple‐ζ basis set [58]. This approach is computationally efficient and well‐suited for large molecular systems while retaining good accuracy in geometries and energetics.

The effect of including dispersion forces was studied by performing calculations using the D3zero, D3BJ, and D4 dispersion‐correction methods by Grimme, Antony, Ehrlich, and Krieg [59, 60, 61, 62]. D3zero represents a zero‐damping version of the D3 method, aimed at correcting dispersion interactions in a computationally efficient manner [59]. D3BJ, an extension of D3, incorporates Becke‐Johnson damping to account for London dispersion energy, particularly in systems where dispersion interactions play a significant role [60]. D4, a more recent development, includes additional higher‐order terms to better capture dispersion energies in complex molecular systems [61, 62]. Each of these methods enhances the description of non‐covalent interactions within Density Functional Theory, thereby improving the accuracy of energy calculations and structural predictions.

It should be noted that not all dispersion corrections are parameterized for each functional employed in this work. Consequently, calculations were conducted using exclusively those functionals designed and implemented with dispersion corrections, as outlined in Table 1. This approach ensures that the coefficients associated with each functional remain consistent, thereby maintaining accuracy and reliability in the results obtained.

All calculations were performed with the large correlation‐consistent def2‐TZVPP basis set, which includes diffuse functions for all atoms [63, 64]. These basis sets treat all electrons for elements H‐Kr and automatically incorporate Stuttgart‐Dresden effective core potentials for elements Rb‐Rn. To consider all electrons, scalar relativistic effects are included using ZORA, with the basis set changed to SARC‐ZORA‐TZVPP for Re and ZORA‐def2‐TZVPP for the remaining elements (H, C, N, O, F, Cl, Mn, and Br). SARC/J was also used [65, 66].

Due to the sensitivity of the Minnesota functionals with the integration grid [67], all calculations were performed with defgrid3, which is a heavier and higher‐quality grid, where the default in the Orca package is defgrid2. The performance of the DFT functionals and dispersion corrections used was assessed by comparison with DLPNO‐CCSD(T) single‐point energies, serving as a high‐level benchmark for relative energy differences [68, 69]. All data obtained and discussed in this work can be found in the Supporting Information.

## 3 Results and Discussion

### 3.1 Geometries and Structural Parameters

**FIGURE 2:**
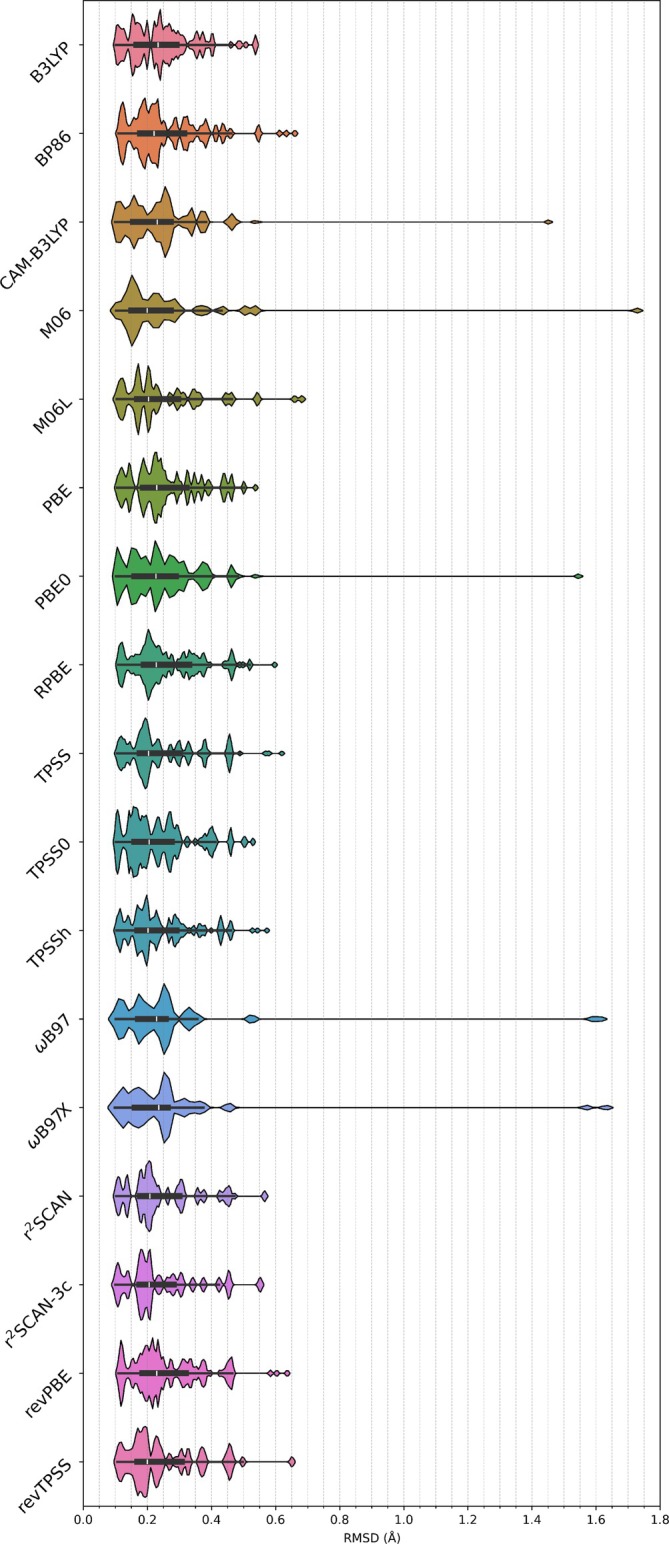
Horizontal violin plots of RMSD values in Å between crystallographic data and optimized structures grouped by functional. The white upright slash denotes the median, the thick black bar signifies the interquartile range, and the thin black line represents the remaining distribution.

The investigation was initiated with an examination of the capacity of the selected functionals and dispersion corrections to precisely describe the geometry in relation to crystallographic data. The calculations were carried out on isolated molecules in the gas phase, thus neglecting intermolecular interactions and environmental effects present in the unit cell. The root mean square deviations (RMSD) for the 34 structures are shown in Figure 2 grouped by functional. The individual functional/dispersion combinations are presented in Figure S1.

In general, the RMSD values range from 0.10–0.70, with only seven functional/dispersion combinations exceeding 0.70 (ranging from 1.45–1.73): CAM–B3LYP, PBE0, ωB97X, ωB97, ωB97(D4), ωB97X(D4), and M06. The observed behavior is indicative of ligand rotation at a carbon–carbon bond in the structure **22** between the aldimine and the anthracene ligands. In fact, with the exception of B3LYP(D3BJ), M06(D3zero), B3LYP(D4), B3LYP, BP86, PBE, revPBE, revTPSS, RPBE, TPSS, TPSS0, and TPSSh, all other methods yielded the highest RMSD values for structure **22**, ranging from 0.46–0.68. These deviations may be attributed to the inherent challenges in accurately modeling the steric effects associated with the ligand rotation in this structure in vacuum. In the unit cell, this compound exhibits π‐stacking between different molecules, whereas in vacuum the molecules are isolated. Consequently, the absence of these interactions forces the torsion angle Re‐N‐CH‐C(anthracene) to shift, as an example, in the optimized structure obtained with ωB97(D4) to −164°, while in the XRD structure it is 172°. This torsion angle variation causes compound **22** to exhibit the largest RMSD values in 42 of the 54 functionals/dispersion combinations evaluated.

Overall, TPSSh, TPSS0, PBE, revPBE, RPBE, r^2^SCAN, TPSS produced optimized structures closest to XRD results without the use of dispersion corrections, with RMSD values ranging from 0.10–0.49. When these functionals are employed with correction dispersion, the RMSD values increased slightly, ranging from 0.50–0.64.

B3LYP, with D3BJ, D3zero, or D4 and without dispersion correction yielded similar results, with RMSD values ranging from 0.10–0.54 in all cases. A similar behavior has been observed for M06L, with D3zero or without dispersion corrections, giving maximum RMSD values of 0.66 and 0.68, respectively.

BP86 without dispersion correction showed a maximum RMSD of 0.55, while the inclusion of D3zero, D3BJ, or D4 increased the maximum RMSD to 0.61, 0,63, and 0.66, respectively. A comparable trend was observed for revTPSS, which presented a maximum RMSD of 0.50 without dispersion and 0.65 when combined with D4.

CAM–B3LYP(D3BJ, D3zero, D4), PBE0(D3zero, D3BJ, D4), and M06(D3zero) performed better with dispersion corrections, reducing the maximum RMSD value by approximately one‐third: From 1.45, 1.55, and 1.73 to 0.48, 0.48, and 0.54, respectively. In contrast, ωB97 and ωB97X with D4 showed RMSD values comparable to those obtained without dispersion corrections, ranging from 1.57–1.63 in both cases. These trends indicate that the efficacy of dispersion corrections in addressing structural deviations is markedly dependent on the underlying functional employed, with some methods benefiting significantly from their inclusion while others show a negligible effect.

Looking at the bond lengths between the metal center and ligands, as well as in the C‐O bonds of the carbonyl groups, slight deviations were observed in comparison to the available experimental data. As shown in Table S1, the mean absolute error (MAE), which is indicative of the average discrepancy between experimental and calculated values, ranges from 0.015 to 0.030, while the standard deviation lies between 0.028 and 0.036, both following the same trend across the different functionals/dispersion combinations.

The lowest MAE value are obtained with TPSS0, both with dispersion corrections (D3BJ, D3zero, D4) and without (0.015–0.016), followed by TPSSh(D3BJ, D3zero, D4, without), PBE0(D3BJ, D3zero, D4, without), CAM–B3LYP(D3BJ, D4), B3LYP(D4), and r^2^SCAN(D3BJ, D4, without), all within the range of 0.016–0.018. This indicates a consistent trend across these methods.

Conversely, RPBE shows the largest deviation, with an MAE of 0.030. When combined with D3zero or D3BJ, the MAE decreases to 0.025, and further to 0.022 with D4. A similar trend is observed for revPBE: Without dispersion correction, the MAE is 0.028, while with D3zero (0.025), D3BJ (0.022), and D4 (0.021), the deviations are reduced. B3LYP also benefits from dispersion correction: Without them, the MAE is 0.025, but decreases to 0.023 (D3zero), 0.019 (D3BJ), and 0.018 (D4).

In the case of ωB97X(D4, without), ωB97(D4, without), and M06L(D3zero, without), no significant differences are observed when the functional is combined with dispersion corrections, with MAEs consistently around 0.025. Similarly, BP86 and PBE show comparable behavior, with MAEs of 0.022 without dispersion corrections and 0.020–0.021 when combined with D3BJ, D3zero, or D4.

For the longest absolute error (ABS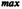), the values range from 0.209 to 0.234. CAM–B3LYP shows the lowest value (0.209), followed by ωB97X (0.211), TPSS0 (0.211–0.212), M06 (0.212), PBE0 (0.213), and ωB97 (0.214). The largest deviations are observed for revPBE and RPBE (0.232–0.234), as well as BP86 (0.229) and PBE (0.229). These values are consistent across both dispersion‐corrected and non‐dispersion‐corrected cases.

Regarding the RMSD analysis, the lowest values are observed for TPSS0, CAM–B3LYP, TPSSh, TPSSh, TPSS, r^2^SCAN, and M06, in the range of 0.028 to 0.030. The highest values are observed for RPBE (0.042), revPBE (0.039), ωB97 (0.039), and M06L (0.038).

The analysis of bond lengths (M‐L and C‐O) shows that TPSS0, TPSSh, and CAM–B3LYP, particularly when combined with dispersion corrections, provide the most accurate geometries with the lowest MAE values and relatively small ABS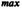 deviations. B3LYP with dispersion corrections and M06 (with or without corrections) also performed well, yielding slightly higher but still competitive deviations. In contrast, RPBE and revPBE consistently produced larger errors, particularly in MAE and RMSD, while ωB97, ωB97X, and M06L, dispersion has little or no impact, and deviations remain significant. These results indicate that, although dispersion corrections often improve structural predictions, their effectiveness strongly depends on the underlying functional.

When considering the overall structural deviations through RMSD analysis, the same trend is observed: TPSS0, TPSSh, CAM–B3LYP, r^2^SCAN, and M06 yield the best agreement with experimental, whereas RPBE, revPBE, ωB97, and M06L perform the worst, with substantially larger RMSD values.

Together, these results indicate that both local (bond‐specific) and global (structural) accuracy are highly sensitive to the functional‐dispersion combination, with TPSS0, TPSSh, and CAM–B3LYP emerging as the most reliable choices across metrics.

### 3.2 Carbonyl Stretching Frequency

It is common practice to obtain Hessians for geometries that are optimized at the same level of theory. This has been the case at least until the single‐point Hessian methodology was developed [70]. Here, we have also performed frequency calculations after geometry optimizations at the selected levels for the benchmark. It is important to note that the carbonyl stretching frequency is directly related to the C‐O bond length, which, due to π‐back‐donation, is influenced by the M‐C interaction [71].

**FIGURE 3:**
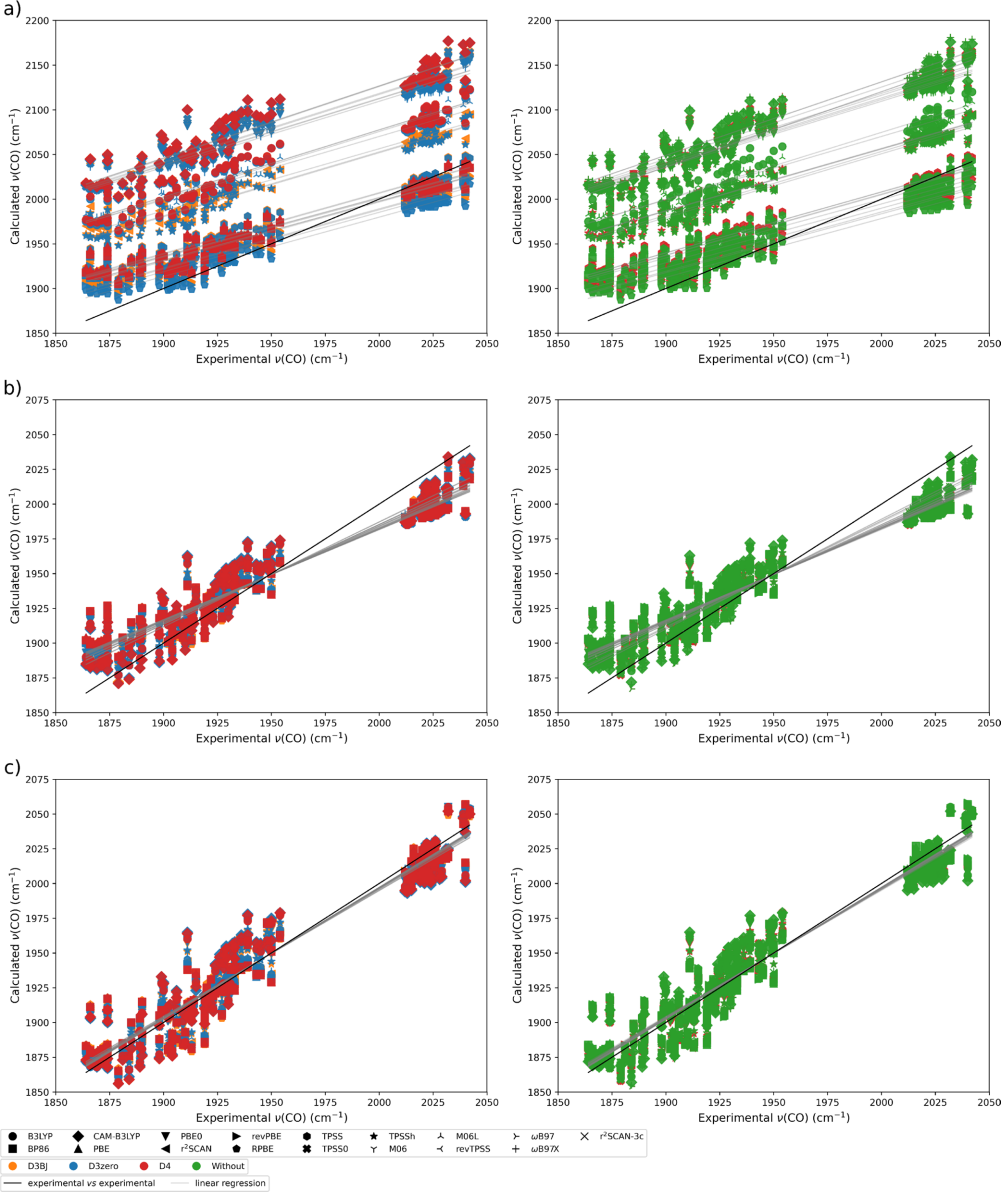
Relation between the calculated and experimental carbonyl stretching frequencies (cm^−1^). (a) Calculated data without correction, (b) calculated data corrected with scale factor, sf, and (c) calculated data corrected with WLS. For better visualization, the data were divided into two plots for each case.

The relation between the experimental and calculated carbonyl stretching frequencies for all compounds, expressed in cm^−1^, is shown in Figure 3a and Table S2, where distinct behaviors for each functional can be clearly observed. The use of dispersion corrections does not have a significant influence on these frequencies, although they do impact the optimization of the structures, as seen in Figures 2, S1, and Table S1.

This finding can be rationalized by the fact that geometry optimizations with and without dispersion do not lead to significantly different bond lengths, particularly in covalently bonded systems. As a result, vibrational frequencies, which depend on bond strengths and atomic masses, may not undergo significant change. Moreover, vibrational modes are localized to bond stretches and bends, whereas dispersion forces are more closely associated with intermolecular or intramolecular non‐covalent interactions.

Initially, we analyzed the distribution of the calculated results across the functional/dispersion combinations using the R^2^ values (Table S2), which fall in the range of 0.90 to 0.95. The lowest value, indicating the highest data dispersion, are observed for CAM–B3LYP with and without dispersion correction (0.90–0.91), followed by ωB97X(D4, without), TPSS0(D3BJ, D3zero, D4, without), B3LYP(D3BJ, D3zero, D4, without), ωB97(D4, without), and M06(D3zero, without), which lie in the range of 0.92 to 0.93. In contrast, r^2^SCAN(D3BJ, D4, without), TPSSh(D4, without), M06L(D3zero, without), r^2^SCAN–3c, TPSS, and TPSSh display lower data dispersion, with higher R^2^ values of 0.95.

However, it is worth noting that ωB97(D4, without), ωB97X(D4, without), and CAM–B3LYP(D3BJ, D3zero, D4, without), despite exhibiting relatively low $R^{2}$ values, also show the largest slopes of the linear regression (0.84–0.80), indicating that these functionals follow the experimental trends more closely. This is followed by TPSS0(D3BJ, D3zero, D4, without) and B3LYP(D3BJ, D3zero, D4, without), with slopes of 0.77–0.76 for TPSS0 and 0.75 for B3LYP, which also have relatively low R^2^ values.

At the opposite end, the lowest slopes are observed for PBE, RPBE, revPBE, and BP86 combined with D3BJ, D3zero, D4, or without dispersion correction, showing values in the range of 0.65–0.66. This is followed by TPSS(D3BJ, D3zero, D4, without), revTPSS(D4, without) and r^2^SCAN(D3BJ, D4, without), and r^2^SCAN‐3c, which exhibit slopes ranging from 0.68–0.70.

In fact, all combinations of functionals and dispersion corrections show slopes less than 1 (the slope of the x=y line), ranging from 0.65–0.84, which reflects a systematic underestimation of the variation in CO stretching frequencies relative to the experimental data. This indicates that, while $R^{2}$ provides a measure of the overall correlation of calculated data, differences between values of 0.95 and 0.90–0.92 already reflect meaningful variations in predictive accuracy. Moreover, the slope must also be considered to fully assess the quality of the fit: Functionals with high $R^{2}$ and slopes closer to unity reproduce both the trend and magnitude of experimental CO stretching frequencies more accurately, whereas functionals with lower $R^{2}$ or slopes further from 1 systematically over‐ or underestimate the frequencies.

Table S3 shows the statistical analysis of the frequency dataset. The lowest values of MAE, ABS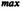, and RMSD are obtained for BP86(D3BJ, D3zero, D4, without), PBE(D3BJ, D3zero, D4, without), revPBE(D3BJ, D3zero, D4, without), revTPSS(D4, without), RPBE(D3BJ, D3zero, D4, without), and TPSS(D3BJ, D3zero, D4, without). This indicates that these functionals yield carbon‐oxygen stretching frequencies with the smallest deviations from the experimental values when each calculated value is compared to its corresponding experimental measurement. In contrast, CAM–B3LYP(D3BJ, D3zero, D4, without), M06(D3zero, without), PBE0(D3BJ, D3zero, D4, without), TPSS0(D3BJ, D3zero, D4, without), ωB97(D4, without), and ωB97X(D4, without) exhibit the opposite behavior, showing the highest values for MAE, ABS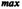, and RMSD.

To quantify the agreement for the investigated systems, we use a deviation coefficient for each functional ($\chi_{func}$, Equation 1), where $\nu_{DFT_{i}}$ is the calculated frequency of complex i, $\nu_{exp_{i}}$ is the experimental value, and $n_{comp}$ is the number of investigated compounds (34). Therefore, the closer the value of $\chi_{func}$ is to zero, the smaller the deviation. The results obtained are presented in Table S3.

(1) $$\begin{array}{l} \chi_{func}=\frac{\sqrt{\sum_{i}{\left(\nu_{DFT_{i}}-\nu_{exp_{i}}\right)}^{2}}}{n_{comp}} \end{array}$$

The trends observed in Figure 3 are confirmed in Table S3, where RPBE(D3BJ, D3zero, D4, without) and revPBE(D3BJ, D3zero, D4, without) exhibited the lowest $\chi_{func}$ values (7 cm^−1^), followed by BP86(D3BJ, D3zero, D4, without) with 8 cm^−1^ and PBE(D3BJ, D3zero, D4, without) with 9 cm^−1^. These results indicate that these functionals produce the smallest deviation of the calculated carbonyl stretching frequencies relative to the experimental values.

On the other hand, CAM–B3LYP(D3BJ, D3zero, D4, without), M06(D3zero, without), PBE0(D3BJ, D3zero, D4, without), TPSS0(D3BJ, D3zero, D4, without), ωB97(D4, without), and ωB97X(D4, without) show the highest deviations (from 37 to 42 cm^−1^), indicating that the calculated frequencies are more distant from the experimental ones.

In general, a functional is capable of producing accurate geometries; however, it often encounters difficulties in stretching frequencies due to limitations in its approach to approximating exchange‐correlation energy, particularly in strongly bonded systems. Since accurate vibrational wavenumbers are dependent on how such functionals deal with electron correlation and anharmonicity effects. The stretching frequencies are known to be susceptible to the potential energy well's asymmetry surrounding the vibrating atoms. DFT frequently encounters limitations in accurately reproducing the asymmetric characteristics of these wells, resulting in frequency inaccuracies.

A common approach to overcome this discrepancy is the use of scale factors (sf), which correct the theoretical vibrational frequencies and bring them closer to experimental values, compensating for two main issues: The approximation inherent to electronic structure calculations and the fact that the potential energy surface is not harmonic, which is more accurately described by the Morse potential in the case of bond stretching vibrations. This correction is obtained using both experimental frequencies $\left(\nu_{exp_{i}}\right)$ and computed values $\left(\nu_{DFT_{i}}\right)$, as shown in Equation (2). The closer the scale factor is to 1, the more similar the calculated and experimental data are.

(2) $$\begin{array}{l} sf=\frac{\sum_{i}(\nu_{exp_{i}}\times \nu_{DFT_{i}})}{\sum_{i}{\left(\nu_{DFT_{i}}\right)}^{2}} \end{array}$$

The obtained scale factors range from 0.93 to 1.00 (Table S3). As expected from the results presented earlier, RPBE and revPBE yielded values equal to unity, while BP86, PBE, revTPSS, and TPSS produced values of 0.99. In contrast, the functionals ωB97X and CAM–B3LYP showed larger deviations (0.93), followed by TPSS0, ωB97, PBE0, and M06 (0.94), in agreement with the trends previously discussed.

When the calculated frequencies are multiplied by the corresponding sf values, it can be observed that, although the overall spread of the data is not reduced, as indicated by the $R^{2}$ values (Table S2), there is a clear convergence between the computed and experimental results (Figure 3b). Similarly, the slope and intercept coefficients show no significant change compared to the uncorrected values (Table S2). However, when comparing the MAE, SD, ABS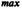, RMSD, and $\chi_{func-sf}$ values (Tables S3 and S4), it is evident that applying a scale factor standardizes the deviations between experimental and calculated values, regardless of the functional or dispersion correction employed.

Another way to correct the discrepancy between calculated and experimental data is through the Wavenumber‐Linear Scaling (WLS) method [72, 73]. The plots of $\nu_{exp}/\nu_{calc}$ versus $\nu_{calc}$ are presented in Figures S2–S10, and the corresponding linear equations are summarized in Table 2. The application of the aforementioned equations to the calculated frequencies yields the data presented in Figure 3c, which exhibits a notable similarity with the experimental values. Although the spread of the values remains unchanged across the three scenarios (Figure 3a–c), the use of WLS leads to slope coefficients closer to unity and intercepts approaching zero (Table S2). This indicates that, after WLS correction, the dataset exhibits an improved agreement between computed and experimental vibrational frequencies, since systematic frequency‐dependent errors inherent in quantum mechanical calculations are corrected, as also reflected in the MAE, SD, ABS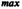, RMSD, and $\chi_{func-WLS}$ values (Table S5).

**TABLE 2:** Linear regression parameters obtained via the Wavenumber‐Linear Scaling method.

Method	Intercept	Slope ($\times 10^{-4}$)
B3LYP(D3BJ)	0.67728	1.36493
BP86(D3BJ)	0.55983	2.20059
CAM–B3LYP(D3BJ)	0.74497	0.90834
PBE(D3BJ)	0.54794	2.24643
PBE0(D3BJ)	0.62063	1.53476
r^2^SCAN(D3BJ)	0.56553	1.97257
revPBE(D3BJ)	0.57099	2.18002
RPBE(D3BJ)	0.57726	2.14368
TPSS(D3BJ)	0.58797	2.02688
TPSS0(D3BJ)	0.67216	1.27461
TPSSh(D3BJ)	0.61298	1.75267
M06(D3zero)	0.62202	1.53723
M06L(D3zero)	0.57982	1.85938
B3LYP(D3zero)	0.68772	1.31886
BP86(D3zero)	0.56484	2.17950
CAM–B3LYP(D3zero)	0.75400	0.86666
PBE(D3zero)	0.54701	2.25456
PBE0(D3zero)	0.62536	1.51411
revPBE(D3zero)	0.57930	2.14857
RPBE(D3zero)	0.58196	2.15467
TPSS(D3zero)	0.59059	2.01668
TPSS0(D3zero)	0.67983	1.24075
TPSSh(D3zero)	0.61781	1.73173
B3LYP(D4)	0.67620	1.36946
BP86(D4)	0.55902	2.20478
CAM–B3LYP(D4)	0.74400	0.91225
PBE(D4)	0.54744	2.24734
PBE0(D4)	0.61980	1.53811
r^2^SCAN(D4)	0.56543	1.97293
revPBE(D4)	0.57346	2.16117
revTPSS(D4)	0.59686	1.97959
RPBE(D4)	0.57971	2.14500
TPSS(D4)	0.58834	2.02263
TPSS0(D4)	0.67075	1.28113
TPSSh(D4)	0.61310	1.75041
ωB97(D4)	0.77648	0.77805
ωB97X(D4)	0.75163	0.86626
r^2^SCAN–3c	0.56900	1.95451
M06	0.61934	1.54991
M06L	0.57960	1.86034
B3LYP	0.67954	1.36154
BP86	0.56328	2.19447
CAM–B3LYP	0.74348	0.91798
PBE	0.54982	2.24367
PBE0	0.61657	1.55795
r^2^SCAN	0.56600	1.97185
revPBE	0.57133	2.19440
revTPSS	0.59774	1.98359
RPBE	0.57670	2.18655
TPSS	0.58807	2.03466
TPSS0	0.67294	1.27634
TPSSh	0.61242	1.76203
ωB97	0.77662	0.77750
ωB97X	0.75253	0.86247

Both correction methods, the scaling factor, sf, and Wavenumber‐Linear Scaling, WLS, allow DFT‐computed results to better match the experimental values. However, it is important to highlight a fundamental difference between them: While the sf approach applies a simple multiplication of the theoretical value by a constant, the WLS method involves a linear equation of the form y=ax+b. This difference becomes evident when comparing Figure 3b,c. With WLS, the trend of calculated ν(CO) values closely overlaps with the experimental data. In contrast, the sf method aligns only the central values, overestimating the lower frequencies and underestimating the higher ones. Consequently, although both methods yield similar statistical indicators, WLS offers a more accurate correction overall.

### 3.3 Hessian Matrix: Negative Eigenvalues

A negative eigenvalue indicates that the optimized geometry corresponds to a saddle point rather than a true minimum. These imaginary modes, derived from the Hessian matrices of the optimized structures, are reported in Table S6 and provide insight into potential instabilities in the computed geometries.

The functional showing the highest occurrence of negative eigenvalues was revTPSS, with twenty out of a total of sixty‐six results that converged to a saddle point. Following this trend, TPSS and M06 each appeared six times; TPSSh, TPSS0, PBE0 and M06L four times; r^2^SCAN, B3LYP and ωB97 three times; RPBE, revPBE, and ωB97X twice; and r^2^SCAN‐3c, PBE, and BP86 once.

Regarding the dispersion corrections, D4 appeared twenty times, D3zero fourteen, D3BJ seven, and calculations without dispersion appeared twenty‐five times. The compounds exhibiting negative eigenvalues were **14** (three occurrences), **15** (six), **18** (thirty‐five), **19** (once), **22** (once), **23** (seven), **26**(two), **28** (four), **30** (four), and **32** (three).

The origin of the negative eigenvalues is mainly associated with rotational or bending motions in the structure, particularly involving the methyl group of acetonitrile. This mode appeared thirty‐five times, representing approximately 53% of all imaginary vibrations, specifically in compound **18**. These frequencies ranged from −57.49 to −2.49 cm^−1^, with the largest magnitude observed for revTPSS(D4) and the smallest for RPBE(D3BJ).

The second most frequent occurrence of imaginary frequencies was also linked to CH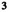 rotation, this time involving the N‐donor ligands. It appeared eighteen times across compounds **23**, **26**, **28**, **30**, and **32**, with values ranging from −98.64 to −1.67 cm^−1^, where the lowest was obtained with M06 and the highest with ωB97(D4).

The remaining cases were associated with CF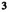 rotation (seven instances), overall bending of the molecular framework (ten instances), and, finally, one case involving phenyl group rotation. It is worth mentioning that in some of these modes, a combination of rotation and bending was also observed, as described in Table S6.

For functionals combined with D4, imaginary frequencies were observed for ωB97, ωB97X, B3LYP, PBE0, r^2^SCAN, revPBE, revTPSS, TPSS, TPSS0 and TPSSh, while they were absent for BP86, CAM–B3LYP, PBE and revPBE. With D3zero, negative eigenvalue appeared for B3LYP, BP86, M06, M06L, PBE, PBE0, revPBE, TPSS, TPSS0, TPSSh, whereas only CAM–B3LYP showed no occurrence. For D3BJ, the functionals that exhibited imaginary frequencies were B3LYP, PBE0, r^2^SCAN, RPBE, TPSS, TPSS0, TPSSh, while BP86, CAM–B3LYP, and PBE displayed none. Finally, in the absence of any dispersion correction, negative eigenvalues were observed for most functionals, except for B3LYP, BP86, CAM–B3LYP, and PBE.

The occurrence of imaginary frequencies in the Hessian matrix further highlights the functional dependence, with hybrid and meta‐GGA functionals such as revTPSS, TPSS, and M06 showing the highest propensity for converging to saddle points, which suggests a sensitivity of these methods to shallow potential energy surfaces. In contrast, global hybrids like PBE0 and B3LYP, despite occasional occurrences, generally exhibited fewer instabilities. Remarkably, CAM–B3LYP did not present any imaginary frequency across all tested schemes (D4, D3BJ, D3zero, and without dispersion), while BP86, revPBE, and PBE only showed negative eigenvalues when combined with D3zero.

It is also crucial to underscore the underlying factors that give rise to the propensity for negative eigenvalues in certain functionals: (i) Approximation limitations: The inherent approximations in the exchange‐correlation functional can induce slight inaccuracies in the potential energy surface, occasionally producing negative curvature along certain vibrational modes. (ii) Numerical errors: Finite numerical precision in geometry optimization and Hessian evaluations may amplify small instabilities, resulting in imaginary frequencies. (iii) Basis set limitations: Incomplete basis sets can inadequately describe electron density in metal‐ligand bonds, which influences Hessian eigenvalues and can promote negative modes. (iv) Reparametrization for transition states: Meta‐GGA and hybrid meta‐GGA functionals are optimized with an emphasis on barrier heights and transition state properties, increasing their tendency to locate geometries near saddle points rather than true minima. Consequently, these factors collectively can explain why these functionals often exhibit a higher occurrence of negative eigenvalues, reflecting both their sensitivity to electronic structure details and the inherent challenges in accurately describing complex coordination geometries.

The predominance of rotational and bending modes, often involving the acetonitrile or ligand CH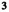 groups, indicates that these negative eigenvalues are not associated with chemically relevant transition states, but rather with low‐energy torsional motions. Consequently, while the presence of imaginary frequencies flags potential limitations in the optimization protocols, their physical impact on the stability of the studied complexes is likely minor. This highlights the importance of carefully balancing functional choice and dispersion correction when modeling such flexible coordination environments.

### 3.4 DLPNO‐CCSD(T) Energies

The accuracy of the selected models (DFT functionals and dispersion corrections combinations) was assessed by performing single‐point DLPNO‐CCSD(T) calculations (default setting, NormalPNO) on the geometries optimized at each DFT level [74]. For each optimized geometry, the corresponding DLPNO‐CCSD(T) single‐point energy was computed, and relative energies (ΔE) were obtained by referencing all structures to the lowest‐energy one. This approach allowed us to determine which functional/dispersion combination provided geometries closer to the high‐level reference minimum. Due to the high computational cost, it was not possible to obtain DLPNO‐CCSD(T) energies for the rhenium‐containing compounds, restricting the analysis to manganese(I) complexes.

**FIGURE 4:**
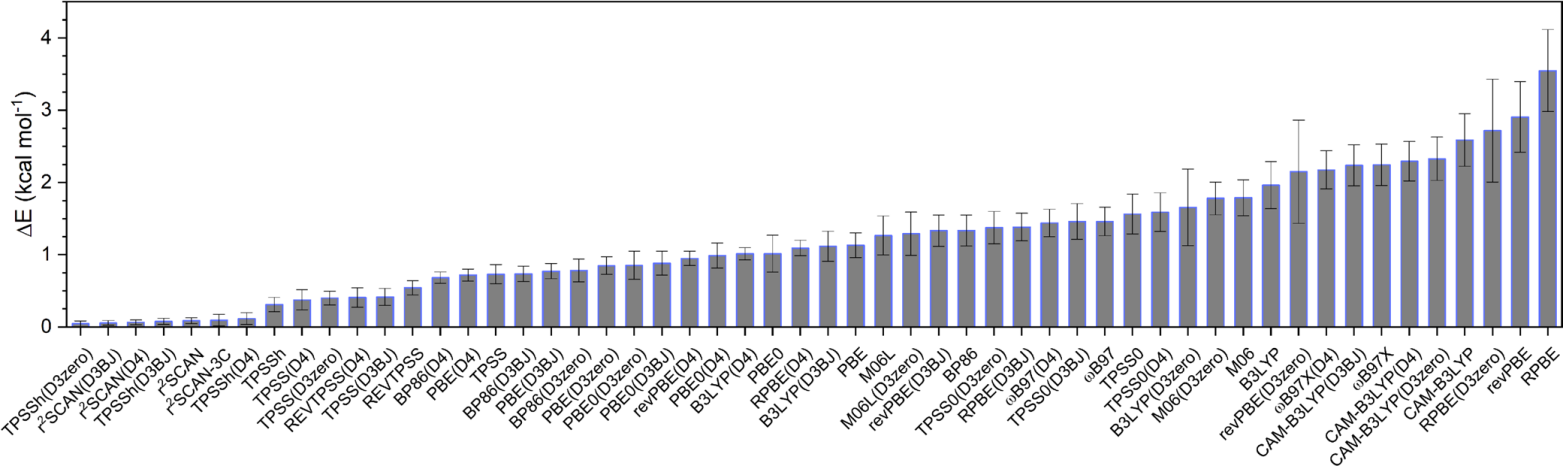
DLPNO‐CCSD(T) relative energies (ΔE) in kcal mol^−1^ obtained from single‐point calculations at geometries optimized with each DFT functional/dispersion combination.

As summarized in Figure 4, the DLPNO‐CCSD(T) relative energies reveal consistent trends in the structural performance of the tested functionals‐dispersion correction combinations. TPSSh(D3zero) provided the most stable geometries across all manganese(I) compounds, with an average ΔE value of 0.04 kcal mol^−1^, followed by r^2^SCAN(D3BJ, D4) with 0.06 kcal mol^−1^. Next in accuracy were TPSSh(D3BJ), r^2^SCAN, r^2^SCAN‐3c, and TPSSh(D4), with ΔE values of 0.08, 0.09, 0.09, and 0.12 kcal mol^−1^, respectively. In contrast, TPSSh without dispersion correction yielded a noticeably larger deviation of 0.31 kcal mol^−1^. These low deviations indicate that such combinations deliver geometries in excellent agreement with the DLPNO‐CCSD(T) reference.

With the exception of TPSS0(D4) and M06L(D3zero), all other functionals produced smaller ΔE values when combined with dispersion correction. For TPSS0, ΔE is 1.56 kcal mol^−1^ without correction and 1.59 kcal mol^−1^ with D4; for M06L, 1.27 kcal mol^−1^ without correction and 1.29 kcal mol^−1^ with D3zero.

Some cases showed negligible differences regardless of wheter dispersion correction was applied, such as ωB97X and ωB97X(D4) (ΔE of 2.24 and 2.17 kcal mol^−1^, respectively); M06 and M06(D3zero) with ΔE of 1.79 and 1.78 kcal mol^−1^; ωB97 and ωB97(D4) with ΔE of 1.46 and 1.49 kcal mol^−1^, indicating that the optimized geometries remain essentially unchanged compared to the DLPNO‐CCSD(T) reference.

For RPBE, dispersion correction significantly improved performance: ΔE decreased from 3.55 kcal mol^−1^ (without correction) to 2.72, 1.18, and 1.09 kcal mol^−1^ with D3zero, D3BJ, and D4, respectively. A similar improvement was observed for revPBE, CAM–B3LYP, B3LYP, TPSS0, BP86, TPSS, revTPSS, and TPSSh, for which the inclusion of dispersion correction consistently yielded structures in closer agreement with the DLPNO‐CCSD(T) reference.

These observations highlight that the inclusion of dispersion corrections does not uniformly improve agreement with high‐level reference; rather, their impact is strongly dependent on the functional and the system under study. For instance, while dispersion significantly improved BP86, B3LYP, and CAM–B3LYP, it had a more modest effect on TPSS0, M06L, ωB97, ωB97X, and M06. This reinforces the need for careful evaluation of dispersion schemes on a case‐by‐case basis rather than assuming a universal benefit.

### 3.5 Cost and Time Efficiency

Selecting a functional and dispersion correction involves not only ensuring accuracy but also considering the computational cost. However, determining precise calculation times can be difficult, as they vary significantly depending not only on the molecular system but also on the computational chemistry software employed and the computational resources available.

**FIGURE 5:**
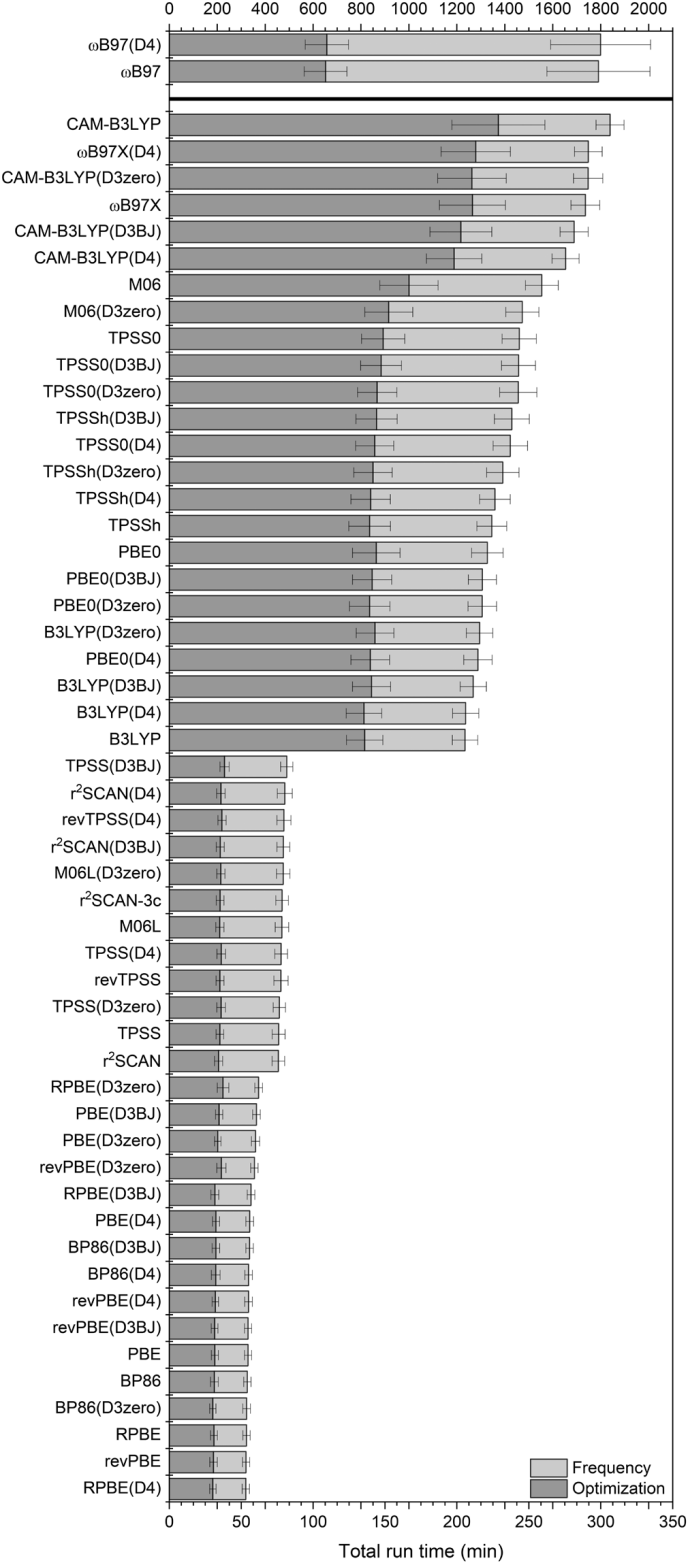
Total run time (in minutes) using 16 processors for each job, separated into optimization and frequency steps.

In this work, all calculations were carried out using the Orca package (version 5.0.4) on the same cluster, utilizing 16 processors with 10 GB of memory each, amounting to a maximum of 160 GB per job. The total calculation times, separated in optimization and frequency steps (Figure 5), were generally unaffected by the use of dispersion correction, as expected, since the increase in computational time due to the inclusion of Grimme's dispersion corrections is generally minimal.

Notably, ωB97 showed the highest computational cost, with maximum run times reaching approximately 150 h, although the values varied considerably, averaging around 30 h. In contrast, the RPBE, revPBE, BP86, and PBE functionals exhibited the shortest calculation times, ranging from 53–62 min. For r^2^SCAN, TPSS, revTPSS, M06L, and r^2^SCAN‐3c, the computational demand was moderate, with run times between 75 to 81 min. Finally, B3LYP, PBE0, TPSSh, TPSS0, M06, CAM–B3LYP, and ωB97X required significantly more time, with average run times ranging from 205–306 min.

The computational times observed in this study align with the anticipated trend, as evidenced by the Jacob's ladder of DFT functionals. Ascending through this ladder typically results in more precise outcomes, though it concomitantly necessitates a substantial escalation in computational demands. Higher‐level functionals, such as ωB97, resulted in significantly longer calculation times. On the other hand, functionals lower on the ladder, such as BP86 and M06L, showed shorter calculation times. These results highlight the inherent balance between computational effort and accuracy: While higher‐level functionals provide more precise electronic descriptions, they come at the expense of increased computational demands. Therefore, it is crucial to consider both the desired accuracy and the available computational resources when selecting a functional.

### 3.6 Overall Assessment of Functionals' Performance

As reported in the geometries and structural parameters section, TPSS, TPSSh, TPSS0, PBE, revPBE, RPBE, and r^2^SCAN provided the most accurate geometrical descriptions compared to X‐ray diffraction data. CAM–B3LYP with dispersion correction (D3zero, D3BJ, and D4) also yielded accurate geometrical results; however, when no dispersion correction was applied, the results were significantly less accurate. For BP86, the opposite behavior was observed, as the inclusion of D3BJ, D3zero, or D4 led to geometries further from the reference. Conversely, ωB97(D4), ωB97X(D4), CAM–B3LYP, ωB97, ωB97X, and M06 produced the largest deviations, indicating limitations in describing coordination environments in metal‐ligand systems.

In the vibrational frequency analysis, especially the carbonyl stretching region, the functionals BP86, PBE, revPBE, revTPSS, RPBE, and TPSS performed best, both with and without dispersion corrections, yielding results closest to the experimental values. In contrast, CAM–B3LYP(D3BJ, D3zero, D4, without), M06(D3zero, without), PBE0(D3BJ, D3zero, D4, without), TPSS0(D3BJ, D3zero, D4, without), ωB97(D4, without), and ωB97X(D4, without) exhibited the largest deviations from experiment. Nevertheless, after applying correction methods (scaling factor and Wavenumber‐Linear Scaling), all combinations yielded calculated stretching frequencies that overlapped with the experimental values.

For the Hessian Matrix, revTPSS exhibited the highest number of negative eigenvalues, followed by TPSSh, TPSS0, PBE0, M06L, r^2^SCAN, B3LYP, ωB97, RPBE, revPBE, ωB97X, r^2^SCAN‐3c, PBE, and BP86. In contrast, CAM–B3LYP did not present negative eigenvalues, while BP86, revPBE, and PBE only showed imaginary frequencies when combined with the D3zero dispersion correction.

Regarding DLPNO‐CCSD(T) single‐point energy calculations on the optimized geometries, TPSSh(D3zero) showed the most stable structures across all combinations, with average ΔE values consistently below 0.04 kcal mol^−1^, followed by r^2^SCAN(D3BJ, D4) with 0.06 kcal mol^−1^ and TPSSh(D3BJ), r^2^SCAN, r^2^SCAN‐3c, and TPSSh(D4), with ΔE values of 0.08, 0.09, 0.09, and 0.12 kcal mol^−1^, respectively. In contrast, RPBE, revPBE, CAM–B3LYP, ωB97X, B3LYP, M06, TPSS0, ωB97, BP86, M06L, PBE, and PBE0, when used without dispersion corrections, displayed the largest deviations, with average ΔE values exceeding 1.00 kcal mol^−1^. Most functionals, however, showed moderate sensitivity to the choice of dispersion correction, leading to a reduction in ΔE compared to the calculations without dispersion correction.

In terms of computational cost, RPBE, revPBE, BP86, and PBE yielded the shortest execution times. The functionals r^2^SCAN, TPSS, revTPSS, M06L, and r^2^SCAN‐3c exhibited a moderate computational cost. All other functionals showed higher computational costs, which remain acceptable considering their consistent accuracy, except for ωB97, which required significantly longer times, up to 150 h in some cases, thereby limiting their practicality.

Taken together, the evaluated parameters revealed that TPSSh, particularly with D3zero correction, yielded geometries that were most stable when assessed by DLPNO‐CCSD(T) single‐point energies, while maintaining consistent accuracy in structural and vibrational predictions. r^2^SCAN with D3BJ or D4 also emerged as a robust option, combining balanced accuracy with moderate computational cost.

On the other hand, CAM–B3LYP with dispersion corrections yielded highly accurate geometries but produced higher relative electronic energies, suggesting deviations from the true energetic minima. Similarly, ωB97 functionals showed significant computational demands, limiting their practicality despite their overall accuracy in certain parameters.

BP86, PBE, and revPBE stood out for their low computational cost and reliable vibrational predictions, but their performance strongly depended on the inclusion of dispersion corrections, without which deviations in both structures and energies became more pronounced.

Overall, these results highlight that no single functional is universally superior; rather, the optimal choice depends on the specific balance required between geometry, vibrational accuracy, energetic stability, and computational efficiency. For metal carbonyl compounds, TPSSh(D3zero) and r^2^SCAN(D3BJ, D4) emerge as the most balanced options. In contrast, CAM–B3LYP excels in geometrical accuracy but fails in energetic stability, whereas BP86 achieves excellent vibration results at low cost but strongly relies on dispersion corrections to remain accurate.

## 4 Conclusions

These findings emphasize the importance of selecting an appropriate functional based on the nature of the system and the desired balance between accuracy and computational cost. In fact, it is imperative to conduct a benchmark to identify the most suitable functional for a particular set of compounds and desired properties. Different functionals are parameterized and optimized based on different sets of data and theoretical constraints. As a result, their performance varies significantly depending on the specific chemical problem under consideration. For instance, a functional that works well for organic molecules might perform poorly for transition metal complexes or systems with significant non‐covalent interactions. Furthermore, the accuracy of a functional is highly dependent on the electronic structure and bonding characteristics of the compounds being studied. Therefore, the presence of lone pairs, highly electronegative atoms, strained rings, or strong electron correlation can challenge different functionals in different ways. Benchmarking allows us to identify the functional that best captures the specific electronic features relevant to your set of compounds, leading to more reliable and meaningful results. The findings reported in this benchmark, summarized in the previous section, guide us in choosing the most appropriate functionals for the adequate description of the properties of metal carbonyl compounds containing nitrogen ligands. These results not only identify the most balanced combinations of accuracy and computational cost for metal carbonyl complexes but also underscore the variability in functional performance depending on the property of interest, be it geometry, vibrational frequencies, or single‐point energies. Future studies may extend these comparisons to a broader range of ligands or metals, as well as explore solvent and dynamic effects to further refine the predictive power of DFT for complex coordination systems.

## Conflicts of Interest

The authors declare no conflicts of interest.

## Supporting information




**Data S1**: Supporting Information.

## Data Availability

The data that support the findings of this study are available in the Supporting Information of this article.

## References

[1] D. A. Kuß , M. Hölscher , and W. Leitner , “Hydrogenation of CO_2_to Methanol With Mn‐PNP‐Pincer Complexes in the Presence of Lewis Acids: The Formate Resting State Unleashed,” ChemCatChem 13, no. 14 (2021): 3319–3323.

[2] M. Guyot , M.‐N. Lalloz , J. S. Aguirre‐Araque , G. Rogez , C. Costentin , and S. Chardon‐Noblat , “Rhenium Carbonyl Molecular Catalysts for CO_2_Electroreduction: Effects on Catalysis of Bipyridine Substituents Mimicking Anchorage Functions to Modify Electrodes,” Inorganic Chemistry 61, no. 40 (2022): 16072–16080. 36166597 10.1021/acs.inorgchem.2c02473

[3] S. Chattopadhyay , M. H. Cheah , R. Lomoth , and L. Hammarström , “Direct Detection of Key Intermediates During the Product Release in Rhenium Bipyridine‐Catalyzed CO_2_ Reduction Reaction,” ACS Catalysis 14, no. 21 (2024): 16324–16334.

[4] J. C. Choate , I. Silva, Jr. , P. C. Hsu , K. Tran , and S. C. Marinescu , “The Positional Effect of an Immobilized re Tricarbonyl Catalyst for CO_2_ Reduction,” ACS Applied Materials & Interfaces 16, no. 38 (2024): 50534–50549. 39255361 10.1021/acsami.4c05536

[5] S. Weber and K. Kirchner , “Manganese Alkyl Carbonyl Complexes: From Iconic Stoichiometric Textbook Reactions to Catalytic Applications,” Accounts of Chemical Research 55, no. 18 (2022): 2740–2751. 36074912 10.1021/acs.accounts.2c00470 PMC9494751

[6] X. Wang , J. Dong , Y. Li , Y. Liu , and Q. Wang , “Visible‐Light‐Mediated Manganese‐Catalyzed Allylation Reactions of Unactivated Alkyl Iodides,” Journal of Organic Chemistry 85, no. 11 (2020): 7459–7467. 32383380 10.1021/acs.joc.0c00861

[7] A. Mandal , M. Pradhan , C. Mitra , S. Nandi , B. Sadhu , and S. Kundu , “Pincer‐(NHC)Mn(I) Complex‐Catalyzed Selective α‐Alkylation of Ketones and Nitriles Using Unactivated Alkenyl Alcohols,” ACS Catalysis 15, no. 2 (2025): 706–718.

[8] H. Liang , Y.‐X. Ji , R.‐H. Wang , Z.‐H. Zhang , and B. Zhang , “Visible‐Light‐Initiated Manganese‐CatalyzedE‐Selective Hydrosilylation and Hydrogermylation of Alkynes,” Organic Letters 21, no. 8 (2019): 2750–2754. 30931573 10.1021/acs.orglett.9b00701

[9] W.‐Z. Weng , H. Liang , R.‐Z. Liu , Y.‐X. Ji , and B. Zhang , “Visible‐Light‐Promoted Manganese‐Catalyzed Atom Transfer Radical Cyclization of Unactivated Alkyl Iodides,” Organic Letters 21, no. 14 (2019): 5586–5590. 31241973 10.1021/acs.orglett.9b01918

[10] L. Wang , J. M. Lear , S. M. Rafferty , S. C. Fosu , and D. A. Nagib , “Ketyl Radical Reactivity via Atom Transfer Catalysis,” Science 362, no. 6411 (2018): 225–229. 30309953 10.1126/science.aau1777 PMC6504239

[11] A. M. Mansour , R. M. Khaled , G. Ferraro , O. R. Shehab , and A. Merlino , “Metal‐Based Carbon Monoxide Releasing Molecules With Promising Cytotoxic Properties,” Dalton Transactions 53 (2024): 9612–9656. 38808485 10.1039/d4dt00087k

[12] A. M. Mansour , R. M. Khaled , and O. R. Shehab , “A Comprehensive Survey of Mn(i) Carbonyls as CO‐Releasing Molecules Reported Over the Last Two Decades,” Dalton Transactions 53 (2024): 19022–19057. 39543968 10.1039/d4dt02091j

[13] E. Stamellou , D. Storz , S. Botov , et al., “Different Design of Enzyme‐Triggered CO‐Releasing Molecules (ET‐CORMs) Reveals Quantitative Differences in Biological Activities in Terms of Toxicity and Inflammation,” Redox Biology 2 (2014): 739–748. 25009775 10.1016/j.redox.2014.06.002 PMC4085349

[14] F. Zobi , A. Degonda , M. C. Schaub , and A. Y. Bogdanova , “CO Releasing Properties and Cytoprotective Effect of Cis‐Trans‐ [ReII(CO)2Br2L2]n Complexes,” Inorganic Chemistry 49, no. 16 (2010): 7313–7322. 20690741 10.1021/ic100458j

[15] P. C. Kunz , H. Meyer , J. Barthel , S. Sollazzo , A. M. Schmidt , and C. Janiak , “Metal Carbonyls Supported on Iron Oxide Nanoparticles to Trigger the CO‐Gasotransmitter Release by Magnetic Heating,” Chemical Communications 49 (2013): 4896–4898. 23609342 10.1039/c3cc41411f

[16] M. S. S. Paqui , V. A. Glitz , D. C. Durigon , et al., “Spectroscopical and Molecular Studies of Four Manganese(I) PhotoCORMs With Bioinspired Ligands Containing Non‐Coordinated Phenol Groups,” Molecules 28, no. 8 (2023): 3439. 37110673 10.3390/molecules28083439 PMC10144837

[17] A. L. Amorim , A. Guerreiro , V. A. Glitz , et al., “Synthesis, Characterization and Photoinduced CO‐Release by Manganese(i) Complexes,” New Journal of Chemistry 44 (2020): 10892–10901.

[18] M. R. Elsby , A. Kumar , L. M. Daniels , et al., “Linear Free Energy Relationships Associated With Hydride Transfer From [(6,6′‐R2‐Bpy)re(CO)3H]: A Cautionary Tale in Identifying Hydrogen Bonding Effects in the Secondary Coordination Sphere,” Inorganic Chemistry 63, no. 41 (2024): 19396–19407. 39344157 10.1021/acs.inorgchem.4c03365

[19] M. Luthra , A. C. Castro , D. Balcells , K. Daasbjerg , and A. Nova , “AI Approaches to Homogeneous Catalysis With Transition Metal Complexes,” ACS Organic & Inorganic Au 5, no. 1 (2025): 26–36. 39927101 10.1021/acsorginorgau.4c00046 PMC11803466

[20] R. F. Cardoso , V. A. Glitz , R. L. T. Parreira , G. F. Caramori , and L. H. S. Lacerda , “The Bonding Situations in Ruthenium Chalcogenonitrosyl Compounds: A Physical Reasoning,” Dalton Transactions 54 (2025): 337–345. 10.1039/d4dt02680b 39544089

[21] W. Hong , M. Luthra , J. B. Jakobsen , et al., “Exploring the Parameters Controlling Product Selectivity in Electrochemical CO2 Reduction in Competition With Hydrogen Evolution Employing Manganese Bipyridine Complexes,” ACS Catalysis 13, no. 5 (2023): 3109–3119. 36910875 10.1021/acscatal.2c05951 PMC9990071

[22] M. C. Colaço , V. A. Glitz , A. K. Jacobs , V. C. Port , and G. F. Caramori , “Supramolecular Chemistry: Exploring the Use of Electronic Structure, Molecular Dynamics, and Machine Learning Approaches,” European Journal of Organic Chemistry 27, no. 27 (2024): e202400367.

[23] G. Frenking , I. Fernández , N. Holzmann , S. Pan , I. Krossing , and M. Zhou , “Metal–CO Bonding in Mononuclear Transition Metal Carbonyl Complexes,” JACS Au 1, no. 5 (2021): 623–645. 34467324 10.1021/jacsau.1c00106 PMC8395605

[24] V. Glitz , D. C. Durigon , A. L. Amorim , et al., “Taming a Silent Killer: Uncovering the Role of Excited States and Uncoordinated Selenium Moieties in the CO Photorelease Mechanism of Manganese(i) Carbonyl Compounds,” Inorganic Chemistry Frontiers 12 (2025): 4677–4690.

[25] K. Sharkas , K. Wagle , B. Santra , et al., “Self‐Interaction Error Overbinds Water Clusters but Cancels in Structural Energy Differences,” Proceedings. National Academy of Sciences. United States of America 117, no. 21 (2020): 11283–11288. 10.1073/pnas.1921258117 PMC7260966 32393631

[26] K. R. Bryenton , A. A. Adeleke , S. G. Dale , and E. R. Johnson , “Delocalization Error: The Greatest Outstanding Challenge in Density‐Functional Theory,” WIREs Computational Molecular Science 13, no. 2 (2023): e1631.

[27] A. Aouina , P. Borlido , M. A. L. Marques , and S. Botti , “Assessing Exchange‐Correlation Functionals for Accurate Densities of Solids,” Journal of Chemical Theory and Computation 20, no. 24 (2024): 10852–10860. 39626866 10.1021/acs.jctc.4c01042 PMC11672669

[28] D. A. Habashy , R. M. Khaled , A. Y. Ahmed , et al., “Cytotoxicity Offac‐Mn(CO)3complexes With a Bidentate Quinoline Ligand Towards Triple Negative Breast Cancer,” Dalton Transactions 51 (2022): 14041–14048. 36106589 10.1039/d2dt01938h

[29] D. X. Ngo , W. W. Kramer , B. J. McNicholas , H. B. Gray , and B. J. Brennan , “Structure, Spectroscopy, and Electrochemistry of Manganese(I) and Rhenium(I) Quinoline Oximes,” Inorganic Chemistry 58, no. 1 (2019): 737–746. 30575373 10.1021/acs.inorgchem.8b02862

[30] S. Gaire , R. J. Ortiz , B. R. Schrage , et al., “(8‐Amino)quinoline and (4‐Amino)phenanthridine Complexes of re(CO)3 Halides,” Journal of Organometallic Chemistry 921 (2020): 121338. 32831401 10.1016/j.jorganchem.2020.121338 PMC7442205

[31] R. Sarkar and K. K. Rajak , “Synthesis and Characterization of Rhenium(I) Complexes Based on O, N, N Coordinating Ligands: DFT/TDDFT Studies on the Electronic Structures and Spectral Properties,” Journal of Organometallic Chemistry 779 (2015): 1–13.

[32] E. F. Tamara Maldonado , D. A. Leonel Llanos , F. G. Andrés Vega , N. A.‐M. Alexis Aspée , and A. G. Guillermo Ferraudi , “Azo–Hydrazone Tautomerism in Organometallic Complexes Triggered by a ‐Re(CO)3(L) Core: A Spectroscopic and Theoretical Study,” Dyes and Pigments 197 (2022): 109953.

[33] K. Ganguli , S. Shee , D. Panja , and S. Kundu , “Cooperative Mn(i)‐Complex Catalyzed Transfer Hydrogenation of Ketones and Imines,” Dalton Transactions 48 (2019): 7358–7366. 30941379 10.1039/c8dt05001e

[34] K. K. R. Tapashi Das , “Synthesis, Characterization and DFT Studies of Complexes Bearing [re(CO)3]+ Core and Reactivity Towards Cyanide Ion,” Journal of Organometallic Chemistry 908 (2020): 121098.

[35] E. Kianfar , U. Monkowius , E. Portenkirchner , and G. Knör , “Synthesis and Characterization of Novel re(BIAN)(CO)3Cl Derivatives Including the First Example of a Water‐Soluble Tricarbonyl Rhenium(I) Complex With Bis(Imino)acenaphthene Ligands,” Zeitschrift für Naturforschung. Teil B 69, no. 6 (2014): 691–698.

[36] G. Knör , M. Leirer , T. E. Keyes , and J. G. Vos , “Non‐Luminescent 1,2‐Diiminetricarbonylrhenium(I) Chloride Complexes – Synthesis, Electrochemical and Spectroscopic Properties of re(DIAN)(CO)3Cl With DIAN =p‐Substituted Bis(Arylimino)acenaphthene,” European Journal of Inorganic Chemistry 2000, no. 4 (2000): 749–751.

[37] J. M. Thomas , P. Vidhyapriya , A. K. Sivan , N. Sakthivel , and C. Sivasankar , “Synthesis, Spectroscopic, CO‐Releasing Ability, and Anticancer Activity Studies of [Mn(CO)3(L–L)Br] Complexes: Experimental and Density Functional Theory Studies,” Applied Organometallic Chemistry 36, no. 6 (2022): e6685.

[38] S. Pordel , B. R. Schrage , C. J. Ziegler , and J. K. White , “Impact of Steric Bulk on Photoinduced Ligand Exchange Reactions in Mn(I) photoCORMs,” Inorganica Chimica Acta 511 (2020): 119845.

[39] A. M. Mansour , C. Steiger , C. Nagel , and U. Schatzschneider , “Wavelength‐Dependent Control of the CO Release Kinetics of Manganese(I) Tricarbonyl PhotoCORMs With Benzimidazole Coligands,” European Journal of Inorganic Chemistry 2019, no. 42 (2019): 4572–4581.

[40] N. Frank , “The ORCA Program System,” WIREs Computational Molecular Science 2, no. 1 (2012): 73–78.

[41] F. Neese , “Software Update: TheORCAprogram System—Version 5.0,” WIREs Computational Molecular Science 12, no. 5 (2022): e1606.

[42] J. P. Perdew , “Density‐Functional Approximation for the Correlation Energy of the Inhomogeneous Electron Gas,” Physical Review B 33 (1986): 8822–8824. 10.1103/physrevb.33.8822 9938299

[43] A. D. Becke , “Density‐Functional Exchange‐Energy Approximation With Correct Asymptotic Behavior,” Physical Review A 38 (1988): 3098–3100. 10.1103/physreva.38.3098 9900728

[44] Y. Zhao and D. G. Truhlar , “A New Local Density Functional for Main‐Group Thermochemistry, Transition Metal Bonding, Thermochemical Kinetics, and Noncovalent Interactions,” Journal of Chemical Physics 125, no. 19 (2006): 194101. 17129083 10.1063/1.2370993

[45] D. G. T. Yan Zhao , “The M06 Suite of Density Functionals for Main Group Thermochemistry, Thermochemical Kinetics, Noncovalent Interactions, Excited States, and Transition Elements: Two New Functionals and Systematic Testing of Four M06‐Class Functionals and 12 Other Functionals,” Theoretical Chemistry Accounts 120, no. 1 (2008): 215–241.

[46] P. J. Stephens , F. J. Devlin , C. F. Chabalowski , and M. J. Frisch , “Ab Initio Calculation of Vibrational Absorption and Circular Dichroism Spectra Using Density Functional Force Fields,” Journal of Physical Chemistry 98, no. 45 (1994): 11623–11627.

[47] T. Yanai , D. P. Tew , and N. C. Handy , “A New Hybrid Exchange–Correlation Functional Using the Coulomb‐Attenuating Method (CAM‐B3LYP),” Chemical Physics Letters 393, no. 1 (2004): 51–57.

[48] J.‐D. Chai and M. Head‐Gordon , “Systematic Optimization of Long‐Range Corrected Hybrid Density Functionals,” Journal of Chemical Physics 128, no. 8 (2008): 084106. 18315032 10.1063/1.2834918

[49] J. P. Perdew , K. Burke , and M. Ernzerhof , “Generalized Gradient Approximation Made Simple,” Physical Review Letters 77, no. 18 (1996): 3865–3868. 10062328 10.1103/PhysRevLett.77.3865

[50] C. Adamo and V. Barone , “Toward Reliable Density Functional Methods Without Adjustable Parameters: The PBE0 Model,” Journal of Chemical Physics 110, no. 13 (1999): 6158–6170.

[51] Y. Zhang and W. Yang , “Comment on “Generalized Gradient Approximation Made Simple”,” Physical Review Letters 80, no. 4 (1998): 890.

[52] B. Hammer , L. B. Hansen , and J. K. Nørskov , “Improved Adsorption Energetics Within Density‐Functional Theory Using Revised Perdew‐Burke‐Ernzerhof Functionals,” Physical Review B 59, no. 11 (1999): 7413–7421.

[53] J. Tao , J. P. Perdew , V. N. Staroverov , and G. E. Scuseria , “Climbing the Density Functional Ladder: Nonempirical Meta–Generalized Gradient Approximation Designed for Molecules and Solids,” Physical Review Letters 91, no. 14 (2003): 146401. 14611541 10.1103/PhysRevLett.91.146401

[54] S. Grimme , “Accurate Calculation of the Heats of Formation for Large Main Group Compounds With Spin‐Component Scaled MP2 Methods,” Journal of Physical Chemistry A 109, no. 13 (2005): 3067–3077. 16833631 10.1021/jp050036j

[55] V. N. Staroverov , G. E. Scuseria , J. Tao , and J. P. Perdew , “Comparative Assessment of a New Nonempirical Density Functional: Molecules and Hydrogen‐Bonded Complexes,” Journal of Chemical Physics 119, no. 23 (2003): 12129–12137. 10.1063/1.4971853 28010100

[56] J. P. Perdew , A. Ruzsinszky , G. I. Csonka , L. A. Constantin , and J. Sun , “Workhorse Semilocal Density Functional for Condensed Matter Physics and Quantum Chemistry,” Physical Review Letters 103, no. 2 (2009): 026403. 19659225 10.1103/PhysRevLett.103.026403

[57] J. W. Furness , A. D. Kaplan , J. Ning , J. P. Perdew , and J. Sun , “Accurate and Numerically Efficient r2SCAN Meta‐Generalized Gradient Approximation,” Journal of Physical Chemistry Letters 11, no. 19 (2020): 8208–8215. 32876454 10.1021/acs.jpclett.0c02405

[58] S. Grimme , A. Hansen , S. Ehlert , and J.‐M. Mewes , “r2SCAN‐3c: A “Swiss Army Knife” Composite Electronic‐Structure Method,” Journal of Chemical Physics 154, no. 6 (2021): 064103. 33588555 10.1063/5.0040021

[59] S. Grimme , J. Antony , S. Ehrlich , and H. Krieg , “A Consistent and Accurateab Initioparametrization of Density Functional Dispersion Correction (DFT‐D) for the 94 Elements H‐Pu,” Journal of Chemical Physics 132, no. 15 (2010): 154104. 20423165 10.1063/1.3382344

[60] S. Grimme , S. Ehrlich , and L. Goerigk , “Effect of the Damping Function in Dispersion Corrected Density Functional Theory,” Journal of Computational Chemistry 32, no. 7 (2011): 1456–1465. 21370243 10.1002/jcc.21759

[61] E. Caldeweyher , C. Bannwarth , and S. Grimme , “Extension of the D3 Dispersion Coefficient Model,” Journal of Chemical Physics 147, no. 3 (2017): 034112. 28734285 10.1063/1.4993215

[62] E. Caldeweyher , S. Ehlert , A. Hansen , et al., “A Generally Applicable Atomic‐Charge Dependent London Dispersion Correction,” Journal of Chemical Physics 150, no. 15 (2019): 154122. 31005066 10.1063/1.5090222

[63] F. Weigend and R. Ahlrichs , “Balanced Basis Sets of Split Valence, Triple Zeta Valence and Quadruple Zeta Valence Quality for H to Rn: Design and Assessment of Accuracy,” Physical Chemistry Chemical Physics 7 (2005): 3297–3305. 16240044 10.1039/b508541a

[64] D. Andrae , U. Häußermann , M. Dolg , H. Stoll , and H. Preuß , “Energy‐Adjustedab Initio Pseudopotentials for the Second and Third Row Transition Elements,” Theoretica Chimica Acta 77 (1990): 123–141.

[65] D. A. Pantazis , X.‐Y. Chen , C. R. Landis , and F. Neese , “All‐Electron Scalar Relativistic Basis Sets for Third‐Row Transition Metal Atoms,” Journal of Chemical Theory and Computation 4 (2008): 908–919. 26621232 10.1021/ct800047t

[66] F. Weigend , “Accurate Coulomb‐Fitting Basis Sets for H to Rn,” Physical Chemistry Chemical Physics 8 (2006): 1057–1065. 16633586 10.1039/b515623h

[67] S. E. Wheeler and K. N. Houk , “Integration Grid Errors for Meta‐GGA‐Predicted Reaction Energies: Origin of Grid Errors for the M06 Suite of Functionals,” Journal of Chemical Theory and Computation 6, no. 2 (2010): 395–404. 20305831 10.1021/ct900639j PMC2840268

[68] C. Riplinger and F. Neese , “An Efficient and Near Linear Scaling Pair Natural Orbital Based Local Coupled Cluster Method,” Journal of Chemical Physics 138, no. 3 (2013): 034106. 23343267 10.1063/1.4773581

[69] C. Riplinger , P. Pinski , U. Becker , E. F. Valeev , and F. Neese , “Sparse Maps—A Systematic Infrastructure for Reduced‐Scaling Electronic Structure Methods. II. Linear Scaling Domain Based Pair Natural Orbital Coupled Cluster Theory,” Journal of Chemical Physics 144, no. 2 (2016): 024109. 26772556 10.1063/1.4939030

[70] S. G. Sebastian Spicher , “Single‐Point Hessian Calculations for Improved Vibrational Frequencies and Rigid‐Rotor‐Harmonic‐Oscillator Thermodynamics,” Journal of Chemical Theory and Computation 17, no. 3 (2021): 1701–1714. 33554604 10.1021/acs.jctc.0c01306

[71] R. K. Hocking and T. W. Hambley , “Database Analysis of Transition Metal Carbonyl Bond Lengths: Insight Into the Periodicity of π Back‐Bonding, σ Donation, and the Factors Affecting the Electronic Structure of the TM−C⋮O Moiety,” Organometallics 26, no. 11 (2007): 2815–2823.

[72] H. Yoshida , A. Ehara , and H. Matsuura , “Density Functional Vibrational Analysis Using Wavenumber‐Linear Scale Factors,” Chemical Physics Letters 325, no. 4 (2000): 477–483.

[73] K. T. Hiroshi Yoshida , A. E. Junko Okamura , and J. Hiroatsu Matsuura , “A New Approach to Vibrational Analysis of Large Molecules by Density Functional Theory: Wavenumber‐Linear Scaling Method,” Physical Chemistry A 106, no. 14 (2002): 3580–3586.

[74] D. G. Liakos and F. Neese , “Is It Possible to Obtain Coupled Cluster Quality Energies at Near Density Functional Theory Cost? Domain‐Based Local Pair Natural Orbital Coupled Cluster vs Modern Density Functional Theory,” Journal of Chemical Theory and Computation 11, no. 9 (2015): 4054–4063. 26575901 10.1021/acs.jctc.5b00359

