# Supplementary material for: Choose Your Level Wisely: Assessing Density Functionals and Dispersion Corrections for Metal Carbonyl Compounds
Source: J Comput Chem. 2025 Oct 10;46(27):e70245. doi: 10.1002/jcc.70245 (PMC12511928; doi:10.1002/jcc.70245)
Supplement: Supplementary file 1 — Data S1: Supporting Information. [file JCC-46-0-s001.zip › Supplementary_information_i.pdf]

Supporting Information: Choose Your Level Wisely: Assessing Density Functionals and Dispersion Corrections for Metal Carbonyl Compounds

Vinícius Glitz,[*,†] Vinícius Capriles Port,[†] Ebbe Nordlander,[‡] Rosely Aparecida Peralta,[†] and Giovanni Finoto Caramori[*,†]

†*Departamento de Química, Universidade Federal de Santa Catarina, Campus Universitário Trindade, 88040-900, Florianópolis, SC, Brasil*

‡*Chemical Physics, Department of Chemistry, Lund University, Box 124, SE-221 00, Lund, Sweden*

E-mail: vinicius.glitz@posgrad.ufsc.br; giovanni.caramori@ufsc.br

List of Figures

S1 Horizontal violin plots of root mean square deviation (RMSD) values between crystallographic data and optimized structures, the white upright slash denotes the median, the thick black bar signifies the interquartile range, and the thin black line represents the remaining distribution. S-4

S2 Ratios of the experimental frequency to the unscaled calculated frequency (ν_{exp}/ν_{calc}) are plotted against the unscaled calculated frequency (ν_{calc}) to obtain the Wavenumber-Linear Scaling (WLS) equation. S-10

S3 Ratios of the experimental frequency to the unscaled calculated frequency (ν_{exp}/ν_{calc}) are plotted against the unscaled calculated frequency (ν_{calc}) to obtain the Wavenumber-Linear Scaling (WLS) equation. S-11

S4 Ratios of the experimental frequency to the unscaled calculated frequency (ν_{exp}/ν_{calc}) are plotted against the unscaled calculated frequency (ν_{calc}) to obtain the Wavenumber-Linear Scaling (WLS) equation. S-12

S5 Ratios of the experimental frequency to the unscaled calculated frequency (ν_{exp}/ν_{calc}) are plotted against the unscaled calculated frequency (ν_{calc}) to obtain the Wavenumber-Linear Scaling (WLS) equation. S-13

S6 Ratios of the experimental frequency to the unscaled calculated frequency (ν_{exp}/ν_{calc}) are plotted against the unscaled calculated frequency (ν_{calc}) to obtain the Wavenumber-Linear Scaling (WLS) equation. S-14

S7 Ratios of the experimental frequency to the unscaled calculated frequency (ν_{exp}/ν_{calc}) are plotted against the unscaled calculated frequency (ν_{calc}) to obtain the Wavenumber-Linear Scaling (WLS) equation. S-15

S8 Ratios of the experimental frequency to the unscaled calculated frequency (ν_{exp}/ν_{calc}) are plotted against the unscaled calculated frequency (ν_{calc}) to obtain the Wavenumber-Linear Scaling (WLS) equation. S-16

S9 Ratios of the experimental frequency to the unscaled calculated frequency (ν_{exp}/ν_{calc}) are plotted against the unscaled calculated frequency (ν_{calc}) to obtain the Wavenumber-Linear Scaling (WLS) equation. S-17
S10 Ratios of the experimental frequency to the unscaled calculated frequency (ν_{exp}/ν_{calc}) are plotted against the unscaled calculated frequency (ν_{calc}) to obtain the Wavenumber-Linear Scaling (WLS) equation. S-18

List of Tables

S1 Mean Absolute Error (MAE), Standard Deviation (SD), Maximum Absolute Error (ABS$_{max}$), and Root Mean Square Deviation (RMSD), for the metal-ligand and carbonyl group bond lengths (Å) across the methods and dispersion corrections combinations used in this work. S-5
S2 Relation between the calculated and experimental carbonyl stretching frequencies (cm^{-1}) for calculated data without correction, calculated data corrected with scale factor (sf), and calculated data corrected with WLS method. S-6
S3 Statistical data (MAE, SD, ABS$_{max}$, and RMSD), scale factors (sf) and the deviation coefficient (χ_{func}) of the carbonyl stretching frequency for the methods and dispersion set. S-7
S4 Scale factors: statistical data (MAE, SD, ABS$_{max}$, and RMSD) and the deviation coefficient ($\chi_{func-sf}$) of the carbonyl stretching frequency for the methods and dispersion set. S-8
S5 Wavenumber-Linear Scaling: statistical data (MAE, SD, ABS$_{max}$, and RMSD) and the deviation coefficient ($\chi_{func-WLS}$) of the carbonyl stretching frequency for the methods and dispersion set. S-9
S6 Imaginary vibrational frequencies (cm^{-1}) and their attributions. S-19

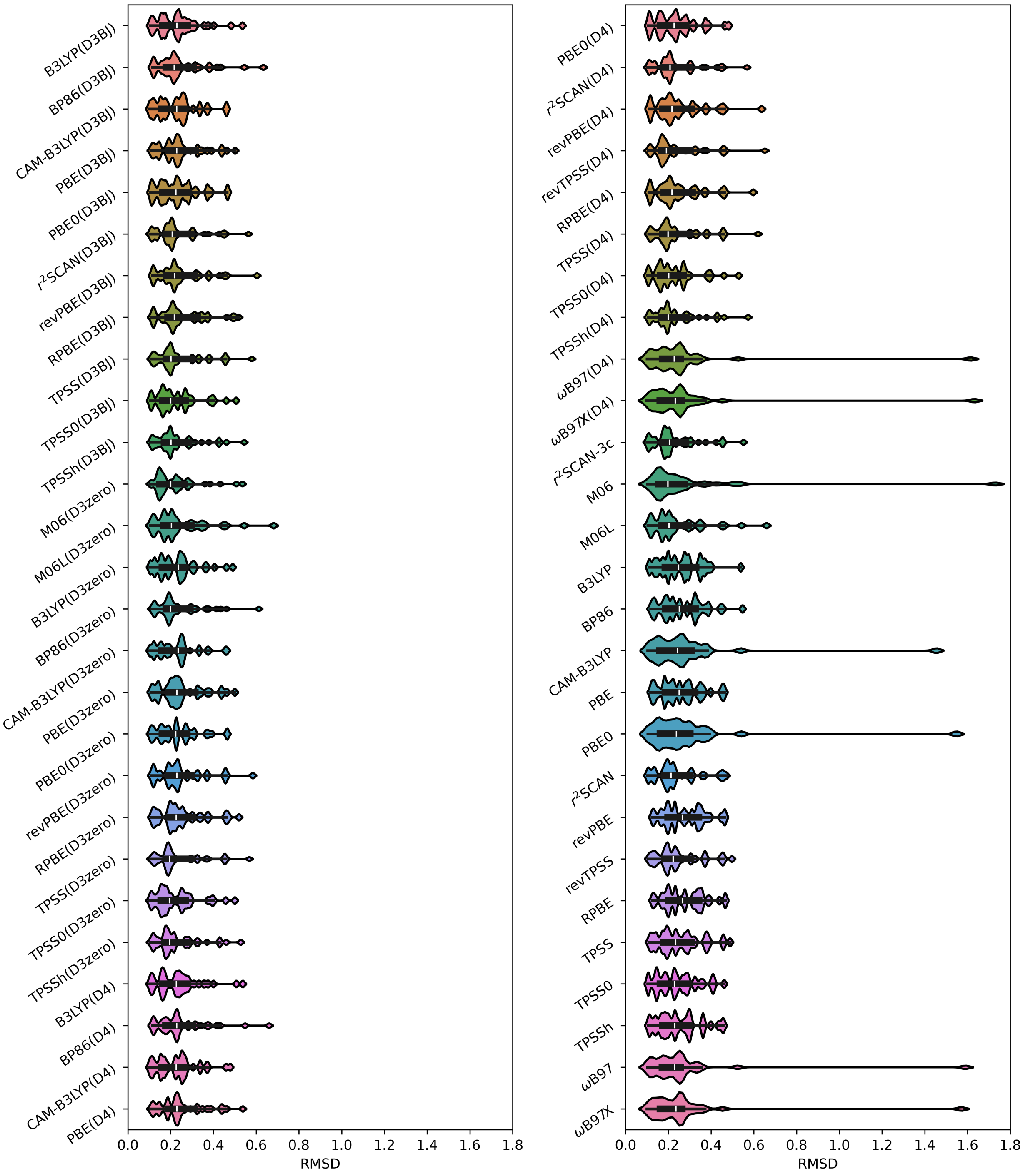

Figure S1: Horizontal violin plots of root mean square deviation (RMSD) values between crystallographic data and optimized structures, the white upright slash denotes the median, the thick black bar signifies the interquartile range, and the thin black line represents the remaining distribution.

Table S1: Mean Absolute Error (MAE), Standard Deviation (SD), Maximum Absolute Error (ABS_{max}), and Root Mean Square Deviation (RMSD), for the metal-ligand and carbonyl group bond lengths (Å) across the methods and dispersion corrections combinations used in this work.

method	SD	ABS_{max}	MAE	RMSD
B3LYP(D3BJ)	0.029	0.216	0.019	0.032
BP86(D3BJ)	0.031	0.229	0.020	0.031
CAM–B3LYP(D3BJ)	0.029	0.209	0.018	0.029
PBE(D3BJ)	0.032	0.229	0.021	0.032
PBE0(D3BJ)	0.029	0.213	0.017	0.029
r^2SCAN(D3BJ)	0.030	0.220	0.018	0.030
revPBE(D3BJ)	0.031	0.232	0.022	0.033
RPBE(D3BJ)	0.035	0.233	0.025	0.035
TPSS(D3BJ)	0.030	0.227	0.019	0.030
TPSS0(D3BJ)	0.028	0.211	0.016	0.028
TPSSh(D3BJ)	0.029	0.220	0.017	0.029
M06(D3zero)	0.029	0.212	0.019	0.030
M06L(D3zero)	0.035	0.220	0.025	0.038
B3LYP(D3zero)	0.033	0.216	0.023	0.037
BP86(D3zero)	0.031	0.229	0.021	0.032
CAM–B3LYP(D3zero)	0.029	0.209	0.019	0.030
PBE(D3zero)	0.032	0.229	0.021	0.032
PBE0(D3zero)	0.029	0.213	0.017	0.029
revPBE(D3zero)	0.034	0.233	0.025	0.038
RPBE(D3zero)	0.034	0.234	0.025	0.038
TPSS(D3zero)	0.029	0.227	0.019	0.030
TPSS0(D3zero)	0.028	0.212	0.015	0.028
TPSSh(D3zero)	0.028	0.221	0.016	0.029
B3LYP(D4)	0.028	0.216	0.018	0.030
BP86(D4)	0.031	0.229	0.020	0.032
CAM–B3LYP(D4)	0.028	0.209	0.017	0.028
PBE(D4)	0.032	0.229	0.021	0.032
PBE0(D4)	0.028	0.213	0.017	0.029
r^2SCAN(D4)	0.030	0.220	0.018	0.030
revPBE(D4)	0.032	0.232	0.021	0.032
revTPSS(D4)	0.032	0.228	0.021	0.032
RPBE(D4)	0.032	0.233	0.022	0.033
TPSS(D4)	0.031	0.227	0.019	0.031
TPSS0(D4)	0.028	0.211	0.016	0.028
TPSSh(D4)	0.029	0.220	0.017	0.029
ωB97(D4)	0.036	0.214	0.025	0.039
ωB97X(D4)	0.034	0.211	0.024	0.037
r^2SCAN–3c	0.031	0.220	0.019	0.031
M06	0.029	0.212	0.019	0.031
M06L	0.035	0.220	0.025	0.038
B3LYP	0.033	0.216	0.025	0.038
BP86	0.031	0.229	0.022	0.033
CAM–B3LYP	0.030	0.209	0.020	0.032
PBE	0.032	0.229	0.022	0.033
PBE0	0.029	0.213	0.017	0.029
r^2SCAN	0.030	0.220	0.018	0.030
revPBE	0.034	0.233	0.028	0.039
revTPSS	0.030	0.228	0.019	0.031
RPBE	0.035	0.234	0.030	0.042
TPSS	0.029	0.227	0.019	0.030
TPSS0	0.028	0.212	0.016	0.028
TPSSh	0.028	0.221	0.017	0.029
ωB97	0.036	0.214	0.025	0.039
ωB97X	0.034	0.211	0.024	0.037

Table S2: Relation between the calculated and experimental carbonyl stretching frequencies (cm^{-1}) for calculated data without correction, calculated data corrected with scale factor (sf), and calculated data corrected with WLS method.

method	without correction			scale factor			WLS		
	slope	intercept	R^2	slope	intercept	R^2	slope	intercept	R^2
B3LYP(D3BJ)	0.75	575	0.93	0.72	550	0.93	0.93	141	0.93
BP86(D3BJ)	0.66	680	0.94	0.65	675	0.94	0.94	115	0.94
CAM–B3LYP(D3BJ)	0.81	515	0.91	0.75	481	0.91	0.91	183	0.91
PBE(D3BJ)	0.65	693	0.94	0.65	686	0.94	0.94	117	0.94
PBE0(D3BJ)	0.74	624	0.94	0.70	585	0.94	0.94	123	0.94
r^2SCAN(D3BJ)	0.69	666	0.95	0.67	643	0.95	0.95	101	0.95
revPBE(D3BJ)	0.66	665	0.94	0.66	664	0.94	0.94	111	0.94
RPBE(D3BJ)	0.66	666	0.94	0.66	665	0.94	0.94	120	0.94
TPSS(D3BJ)	0.68	649	0.94	0.67	640	0.95	0.95	104	0.95
TPSS0(D3BJ)	0.77	586	0.92	0.72	551	0.92	0.92	152	0.92
TPSSh(D3BJ)	0.72	622	0.95	0.69	602	0.95	0.95	107	0.95
M06(D3zero)	0.74	633	0.93	0.69	595	0.93	0.93	133	0.93
M06L(D3zero)	0.71	651	0.95	0.68	624	0.95	0.95	96	0.95
B3LYP(D3zero)	0.75	572	0.92	0.72	548	0.92	0.92	150	0.92
BP86(D3zero)	0.66	670	0.94	0.66	666	0.94	0.94	110	0.94
CAM–B3LYP(D3zero)	0.81	506	0.90	0.76	477	0.90	0.90	188	0.90
PBE(D3zero)	0.65	693	0.94	0.65	686	0.94	0.94	117	0.94
PBE0(D3zero)	0.75	619	0.94	0.70	587	0.93	0.94	126	0.94
revPBE(D3zero)	0.66	656	0.94	0.66	654	0.94	0.94	109	0.94
RPBE(D3zero)	0.66	653	0.94	0.66	655	0.94	0.94	109	0.94
TPSS(D3zero)	0.68	644	0.94	0.67	638	0.95	0.94	107	0.95
TPSS0(D3zero)	0.77	585	0.92	0.72	546	0.92	0.92	162	0.92
TPSSh(D3zero)	0.72	621	0.94	0.69	599	0.94	0.94	110	0.94
B3LYP(D4)	0.75	576	0.93	0.72	550	0.93	0.93	139	0.93
BP86(D4)	0.66	679	0.94	0.65	675	0.94	0.94	115	0.94
CAM–B3LYP(D4)	0.81	513	0.91	0.75	482	0.91	0.91	182	0.91
PBE(D4)	0.66	691	0.94	0.65	686	0.94	0.94	116	0.94
PBE0(D4)	0.74	625	0.94	0.70	586	0.94	0.94	123	0.94
r^2SCAN(D4)	0.69	667	0.95	0.67	643	0.95	0.95	101	0.95
revPBE(D4)	0.66	663	0.94	0.66	661	0.94	0.94	111	0.94
revTPSS(D4)	0.68	641	0.94	0.68	633	0.94	0.94	109	0.94
RPBE(D4)	0.66	658	0.94	0.66	657	0.94	0.94	110	0.94
TPSS(D4)	0.68	648	0.94	0.67	637	0.94	0.95	106	0.95
TPSS0(D4)	0.77	587	0.92	0.72	552	0.92	0.92	152	0.92
TPSSh(D4)	0.72	623	0.95	0.69	602	0.95	0.94	107	0.95
ωB97(D4)	0.84	435	0.93	0.79	408	0.93	0.93	142	0.93
ωB97X(D4)	0.82	487	0.92	0.77	455	0.92	0.92	163	0.92
r^2SCAN–3c	0.70	663	0.95	0.67	641	0.95	0.95	100	0.95
M06	0.74	633	0.93	0.69	596	0.93	0.93	131	0.93
M06L	0.71	651	0.95	0.68	623	0.95	0.95	97	0.95
B3LYP	0.75	576	0.93	0.72	550	0.93	0.93	145	0.93
BP86	0.66	678	0.94	0.65	672	0.94	0.94	117	0.94
CAM–B3LYP	0.80	516	0.91	0.75	483	0.91	0.91	182	0.91
PBE	0.65	690	0.94	0.65	683	0.94	0.94	118	0.94
PBE0	0.74	628	0.94	0.70	589	0.94	0.94	120	0.94
r^2SCAN	0.70	666	0.95	0.67	643	0.95	0.95	100	0.95
revPBE	0.66	667	0.94	0.66	668	0.94	0.94	114	0.94
revTPSS	0.68	640	0.94	0.67	634	0.94	0.94	110	0.94
RPBE	0.66	663	0.94	0.66	664	0.94	0.94	113	0.94
TPSS	0.68	647	0.95	0.67	640	0.94	0.94	108	0.94
TPSS0	0.76	588	0.92	0.72	552	0.92	0.92	156	0.92
TPSSh	0.71	623	0.95	0.69	602	0.95	0.95	106	0.95
ωB97	0.84	435	0.93	0.79	408	0.93	0.93	142	0.93
ωB97X	0.82	488	0.92	0.77	454	0.92	0.92	164	0.92

Table S3: Statistical data (MAE, SD, ABS_{max}, and RMSD), scale factors (sf) and the deviation coefficient (χ_{func}) of the carbonyl stretching frequency for the methods and dispersion set.

method	MAE	SD	ABS_{max}	RMSD	χ_{func}	sf	$\chi_{func-sf}$	$\chi_{func-WLS}$
B3LYP(D3BJ)	91	19	137	93	27	0.96	6	5
BP86(D3BJ)	22	22	72	27	8	0.99	7	4
CAM–B3LYP(D3BJ)	137	19	189	138	41	0.93	6	5
PBE(D3BJ)	24	22	77	30	9	0.99	7	4
PBE0(D3BJ)	126	19	174	128	38	0.94	6	4
r^2SCAN(D3BJ)	73	20	126	75	22	0.96	6	4
revPBE(D3BJ)	19	22	62	23	7	1.00	7	4
RPBE(D3BJ)	20	22	64	23	7	1.00	7	4
TPSS(D3BJ)	26	21	79	32	10	0.99	6	4
TPSS0(D3BJ)	131	19	179	132	39	0.94	6	5
TPSSh(D3BJ)	68	19	119	71	21	0.97	6	4
M06(D3zero)	123	19	171	125	37	0.94	6	4
M06L(D3zero)	86	19	138	88	26	0.96	6	4
B3LYP(D3zero)	89	19	137	91	27	0.96	6	5
BP86(D3zero)	22	22	70	26	8	0.99	7	4
CAM–B3LYP(D3zero)	136	19	189	137	41	0.93	6	5
PBE(D3zero)	24	22	76	29	9	0.99	7	4
PBE0(D3zero)	126	19	173	127	38	0.94	6	4
revPBE(D3zero)	19	22	59	22	7	1.00	6	4
RPBE(D3zero)	18	22	54	22	7	1.00	6	4
TPSS(D3zero)	25	21	78	31	9	0.99	6	4
TPSS0(D3zero)	130	19	179	131	39	0.94	6	5
TPSSh(D3zero)	67	19	118	70	21	0.97	6	4
B3LYP(D4)	91	19	137	93	28	0.96	6	5
BP86(D4)	22	22	72	27	8	0.99	7	4
CAM–B3LYP(D4)	137	19	189	138	41	0.93	6	5
PBE(D4)	24	22	77	30	9	0.99	7	4
PBE0(D4)	127	19	174	128	38	0.94	6	4
r^2SCAN(D4)	73	20	126	75	22	0.96	6	4
revPBE(D4)	20	22	64	23	7	1.00	7	4
revTPSS(D4)	26	21	80	32	10	0.99	6	4
RPBE(D4)	19	22	60	22	7	1.00	6	4
TPSS(D4)	26	21	79	33	10	0.99	6	4
TPSS0(D4)	131	19	179	132	39	0.94	6	5
TPSSh(D4)	68	19	119	71	21	0.97	6	4
ωB97(D4)	129	16	176	130	38	0.94	5	5
ωB97X(D4)	141	18	191	142	42	0.93	6	5
r^2SCAN–3c	73	20	127	76	22	0.96	6	4
M06	123	19	171	125	37	0.94	6	4
M06L	86	19	138	88	26	0.96	6	4
B3LYP	88	19	136	90	27	0.96	6	5
BP86	21	22	69	26	8	0.99	7	4
CAM–B3LYP	136	19	188	137	41	0.93	6	5
PBE	23	22	75	29	9	0.99	7	4
PBE0	125	19	173	127	38	0.94	6	4
r^2SCAN	72	20	126	75	22	0.96	6	4
revPBE	19	22	59	22	7	1.00	7	4
revTPSS	25	21	78	31	9	0.99	6	4
RPBE	18	22	53	22	7	1.00	7	4
TPSS	24	21	77	30	9	0.99	6	4
TPSS0	129	19	178	130	39	0.94	6	5
TPSSh	66	19	117	69	20	0.97	6	4
ωB97	128	16	176	130	38	0.94	5	5
ωB97X	141	18	191	142	42	0.93	6	5

Table S4: Scale factors: statistical data (MAE, SD, ABS_{max}, and RMSD) and the deviation coefficient ($\chi_{func-sf}$) of the carbonyl stretching frequency for the methods and dispersion set.

method	SD	ABS_{max}	MAE	RMSD	$\chi_{func-sf}$
B3LYP(D3BJ)	20	48	17	20	6
BP86(D3BJ)	22	57	19	22	7
CAM-B3LYP(D3BJ)	20	51	17	20	6
PBE(D3BJ)	23	57	19	23	7
PBE0(D3BJ)	21	50	17	21	6
r^2SCAN(D3BJ)	21	55	18	21	6
revPBE(D3BJ)	22	57	18	22	7
RPBE(D3BJ)	22	57	19	22	7
TPSS(D3BJ)	21	55	18	21	6
TPSS0(D3BJ)	20	49	17	20	6
TPSSh(D3BJ)	20	52	17	20	6
M06(D3zero)	21	49	18	21	6
M06L(D3zero)	21	54	17	21	6
B3LYP(D3zero)	20	49	17	20	6
BP86(D3zero)	22	56	18	22	7
CAM-B3LYP(D3zero)	20	52	17	20	6
PBE(D3zero)	23	57	19	23	7
PBE0(D3zero)	21	50	17	21	6
revPBE(D3zero)	22	56	18	22	6
RPBE(D3zero)	22	56	18	22	6
TPSS(D3zero)	21	55	18	21	6
TPSS0(D3zero)	21	49	17	21	6
TPSSh(D3zero)	20	52	17	20	6
B3LYP(D4)	20	48	17	20	6
BP86(D4)	22	57	19	22	7
CAM-B3LYP(D4)	20	51	17	20	6
PBE(D4)	23	57	19	23	7
PBE0(D4)	21	50	17	21	6
r^2SCAN(D4)	21	55	18	21	6
revPBE(D4)	22	56	18	22	7
REVTPSS(D4)	21	56	18	21	6
RPBE(D4)	22	57	18	22	6
TPSS(D4)	21	55	18	21	6
TPSS0(D4)	20	48	17	20	6
TPSSh(D4)	20	52	17	20	6
wB97(D4)	18	47	15	18	5
ωB97X(D4)	19	50	16	19	6
r^2SCAN3C	21	56	18	21	6
M06	21	49	18	21	6
M06L	21	54	17	21	6
B3LYP	20	48	17	20	6
BP86	22	57	19	22	7
CAM-B3LYP	20	52	17	20	6
PBE	23	58	19	23	7
PBE0	21	50	17	21	6
r^2SCAN	21	55	18	21	6
revPBE	22	57	18	22	7
REVTPSS	21	56	18	21	6
RPBE	22	57	18	22	7
TPSS	21	56	18	21	6
TPSS0	21	49	17	21	6
TPSSh	20	53	17	20	6
ωB97	18	47	15	18	5
ωB97X	19	50	16	19	6

Table S5: Wavenumber-Linear Scaling: statistical data (MAE, SD, ABS_{max}, and RMSD) and the deviation coefficient ($\chi_{func-WLS}$) of the carbonyl stretching frequency for the methods and dispersion set.

method	SD	ABS_{max}	MAE	RMSD	$\chi_{func-sf}$
B3LYP(D3BJ)	16	49	13	16	5
BP86(D3BJ)	14	45	11	14	4
CAM-B3LYP(D3BJ)	18	53	14	18	5
PBE(D3BJ)	14	46	11	14	4
PBE0(D3BJ)	15	46	12	15	4
r^2SCAN(D3BJ)	13	44	10	13	4
revPBE(D3BJ)	14	45	10	14	4
RPBE(D3BJ)	14	47	11	14	4
TPSS(D3BJ)	14	44	10	14	4
TPSS0(D3BJ)	16	51	13	16	5
TPSSh(D3BJ)	14	41	11	14	4
M06(D3zero)	15	51	12	15	4
M06L(D3zero)	13	43	10	13	4
B3LYP(D3zero)	16	51	13	16	5
BP86(D3zero)	14	45	11	14	4
CAM-B3LYP(D3zero)	18	54	15	18	5
PBE(D3zero)	14	46	11	14	4
PBE0(D3zero)	15	47	12	15	4
revPBE(D3zero)	14	45	10	14	4
RPBE(D3zero)	14	45	10	14	4
TPSS(D3zero)	14	44	10	14	4
TPSS0(D3zero)	17	52	13	17	5
TPSSh(D3zero)	14	41	11	14	4
B3LYP(D4)	16	49	12	16	5
BP86(D4)	14	45	11	14	4
CAM-B3LYP(D4)	18	53	14	18	5
PBE(D4)	14	46	11	14	4
PBE0(D4)	14	46	11	14	4
r^2SCAN(D4)	13	44	10	13	4
revPBE(D4)	14	45	10	14	4
REVTPSS(D4)	14	45	10	14	4
RPBE(D4)	14	46	10	14	4
TPSS(D4)	14	44	10	14	4
TPSS0(D4)	16	51	13	16	5
TPSSh(D4)	14	41	11	14	4
wB97(D4)	16	48	12	16	5
ωB97X(D4)	17	52	14	17	5
r^2SCAN3C	13	45	10	13	4
M06	15	50	12	15	4
M06L	13	43	10	13	4
B3LYP	16	51	13	16	5
BP86	14	46	11	14	4
CAM-B3LYP	18	54	14	18	5
PBE	14	47	11	14	4
PBE0	14	47	11	14	4
r^2SCAN	13	44	10	13	4
revPBE	14	47	11	14	4
REVTPSS	14	45	10	14	4
RPBE	14	47	11	14	4
TPSS	14	45	10	14	4
TPSS0	16	52	13	16	5
TPSSh	14	42	10	14	4
ωB97	16	48	12	16	5
ωB97X	17	52	14	17	5

(a)

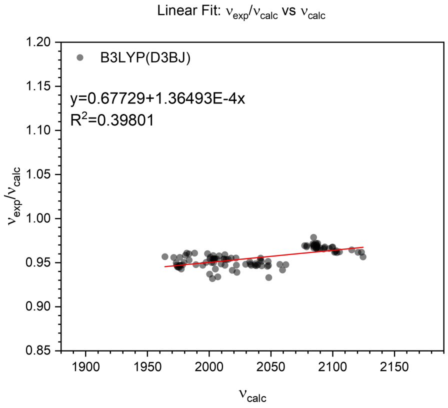

(b)

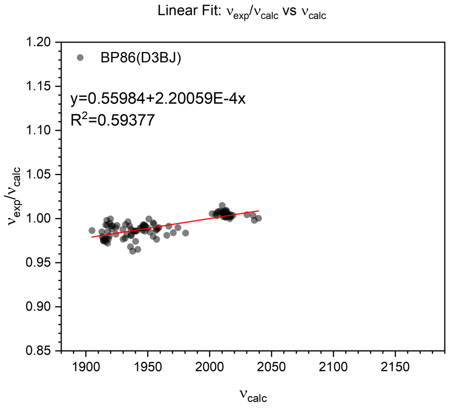

(c)

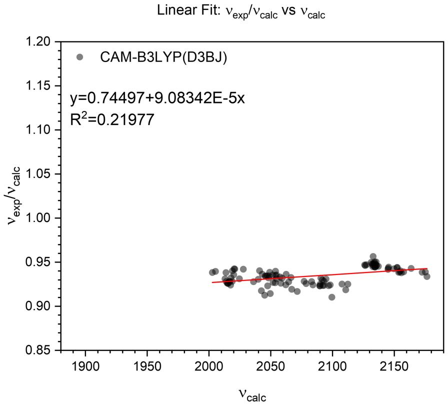

(d)

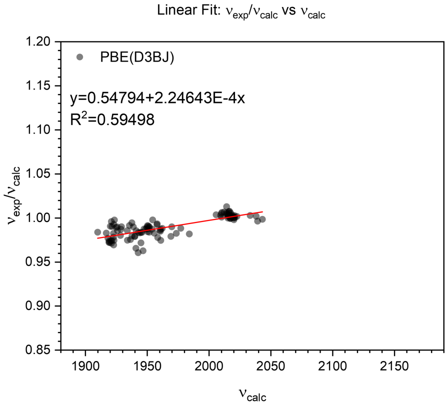

(e)

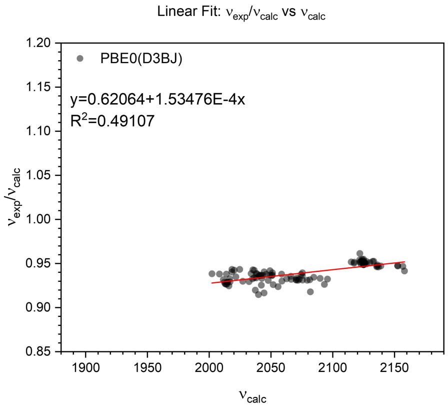

(f)

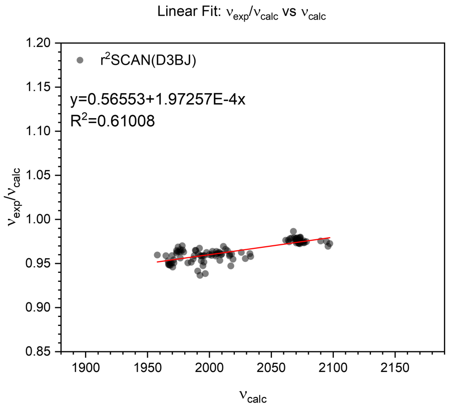

Figure S2: Ratios of the experimental frequency to the unscaled calculated frequency (ν_{exp}/ν_{calc}) are plotted against the unscaled calculated frequency (ν_{calc}) to obtain the Wavenumber-Linear Scaling (WLS) equation.

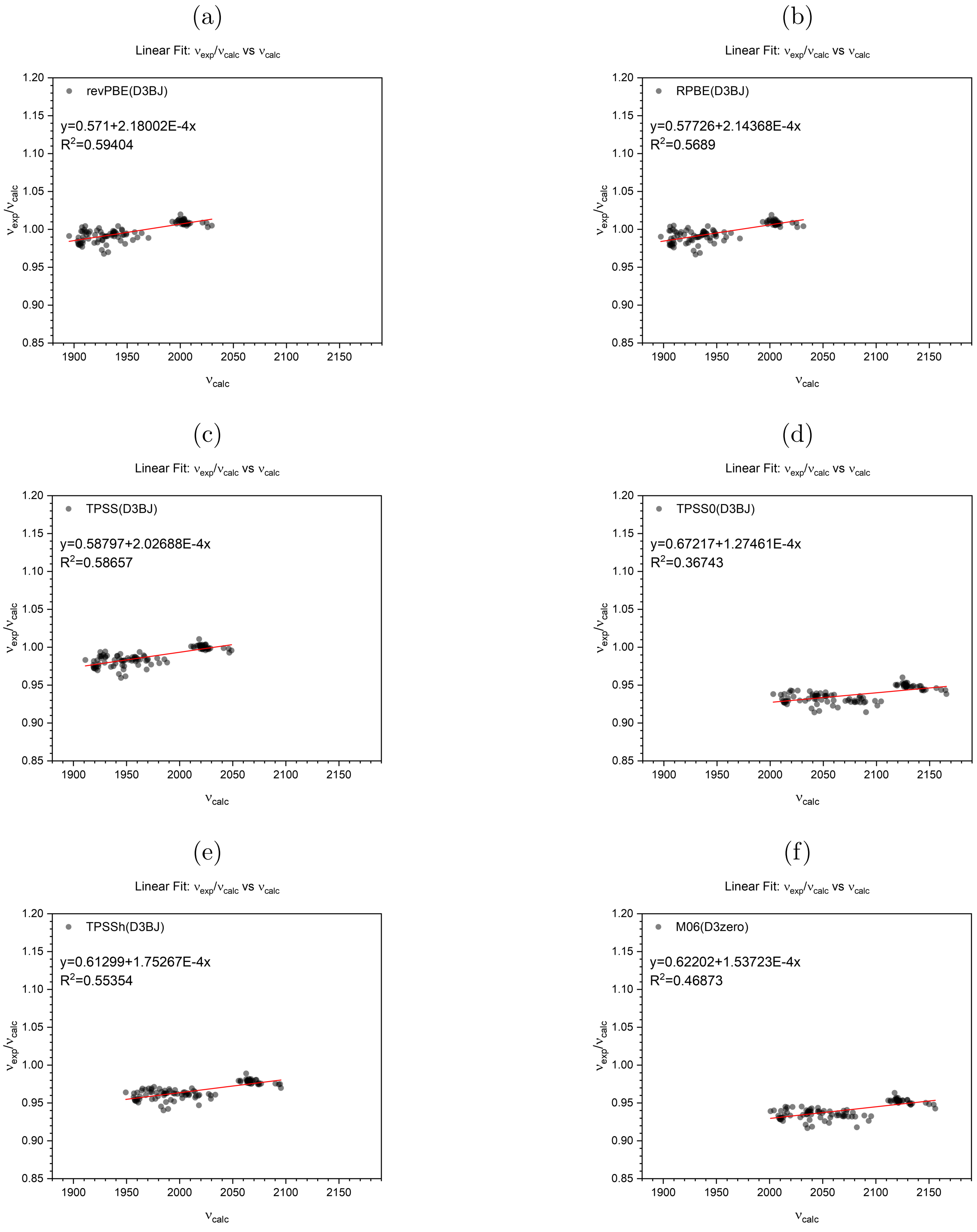

Figure S3: Ratios of the experimental frequency to the unscaled calculated frequency (ν_{exp}/ν_{calc}) are plotted against the unscaled calculated frequency (ν_{calc}) to obtain the Wavenumber-Linear Scaling (WLS) equation.

(a)

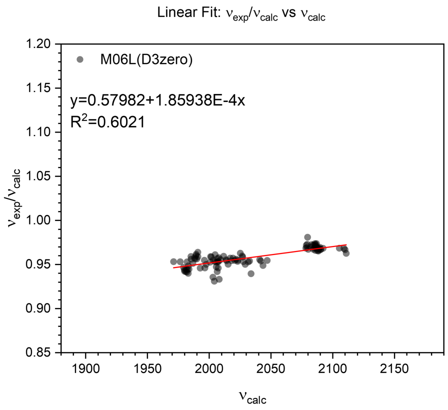

(b)

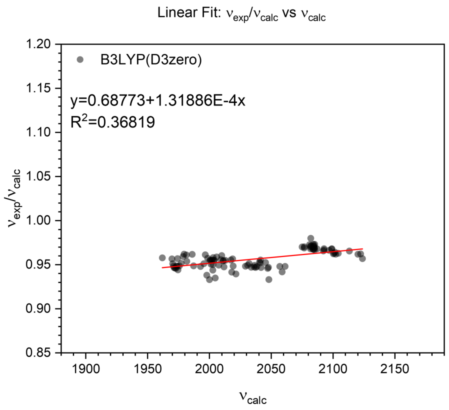

(c)

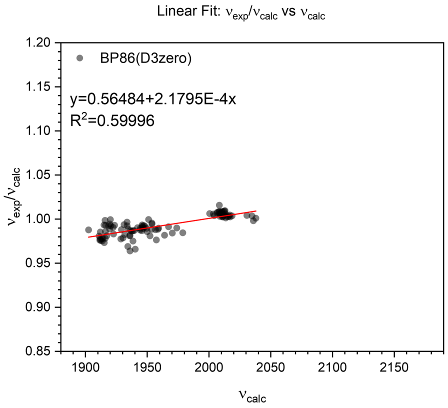

(d)

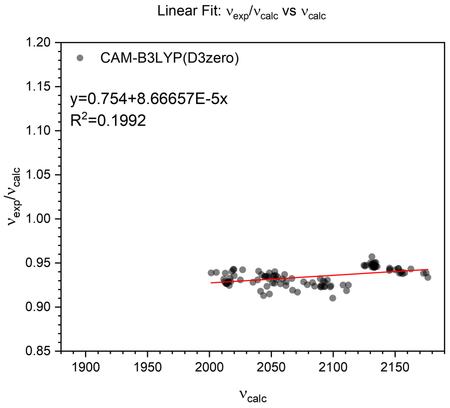

(e)

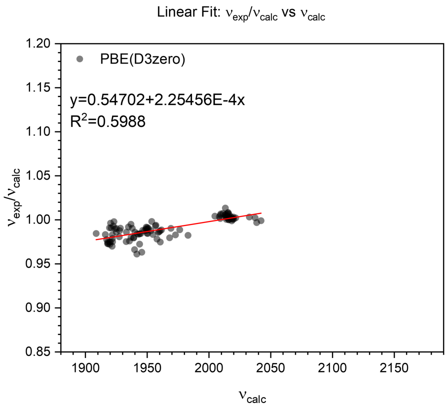

(f)

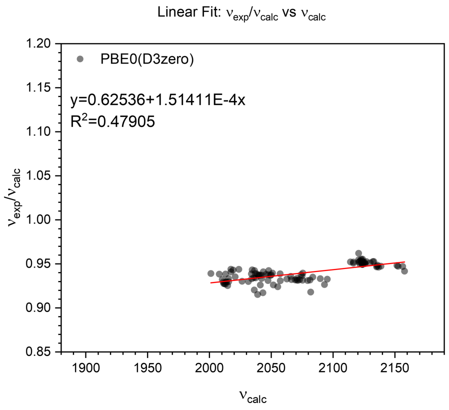

Figure S4: Ratios of the experimental frequency to the unscaled calculated frequency (ν_{exp}/ν_{calc}) are plotted against the unscaled calculated frequency (ν_{calc}) to obtain the Wavenumber-Linear Scaling (WLS) equation.

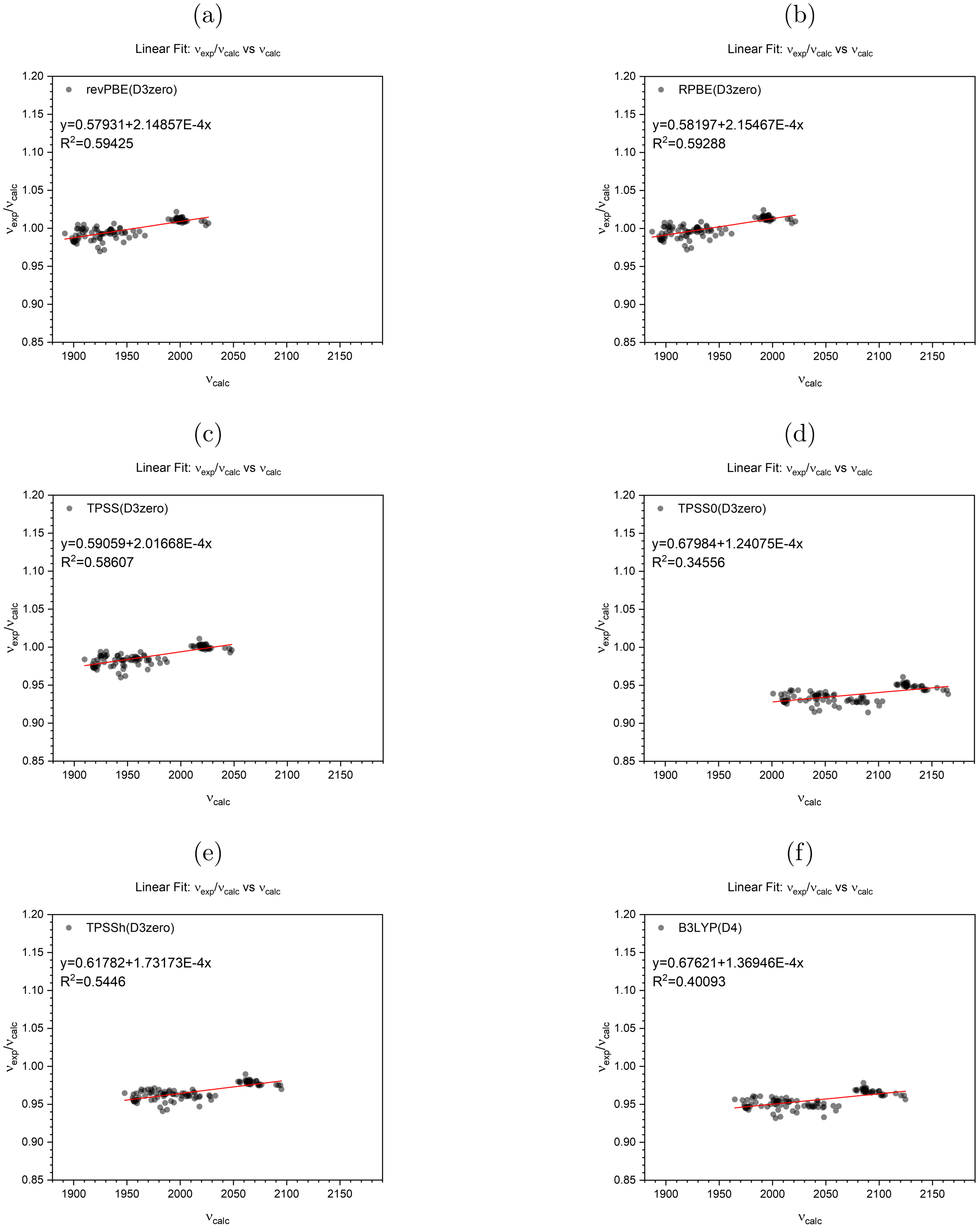

Figure S5: Ratios of the experimental frequency to the unscaled calculated frequency (ν_{exp}/ν_{calc}) are plotted against the unscaled calculated frequency (ν_{calc}) to obtain the Wavenumber-Linear Scaling (WLS) equation.

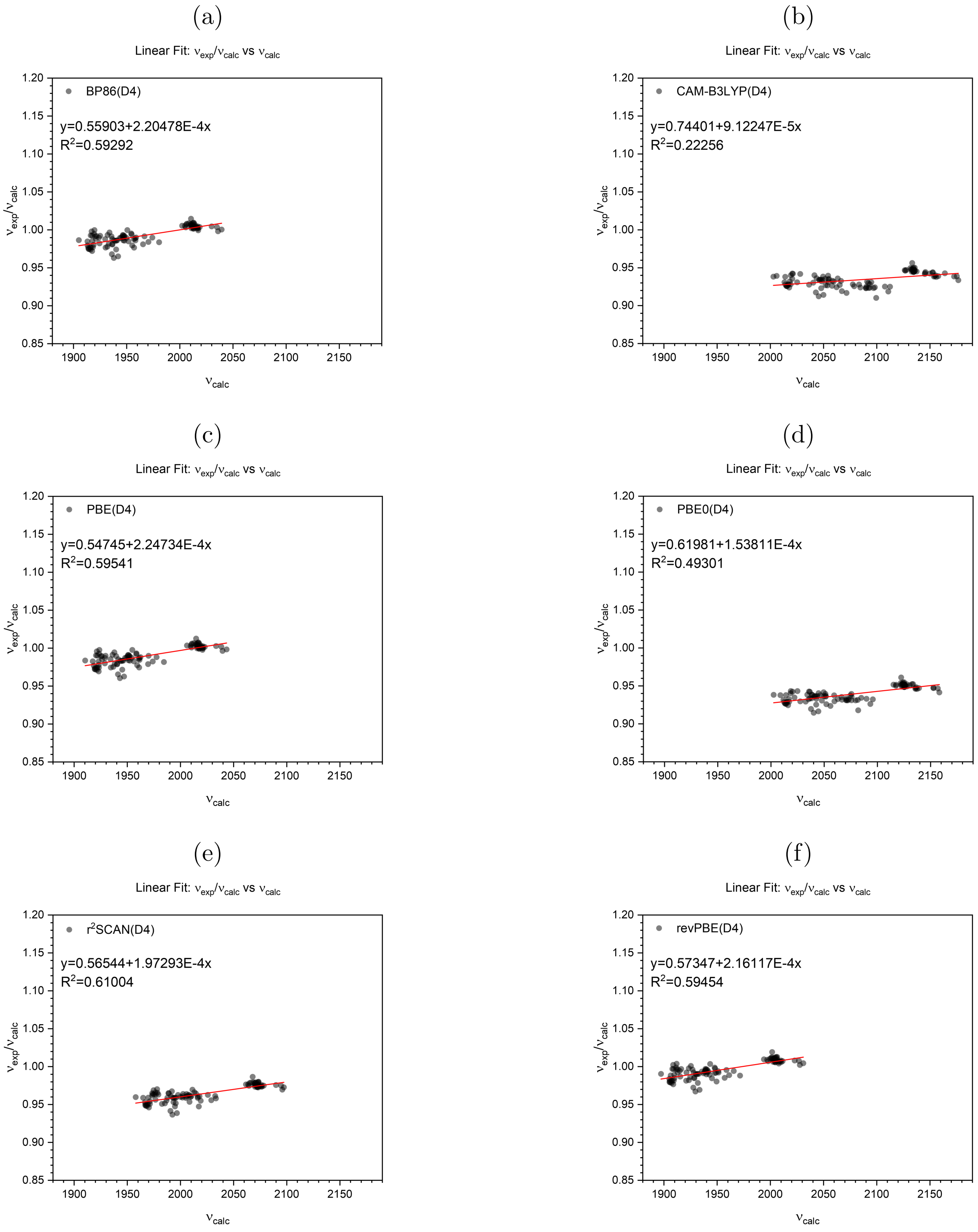

Figure S6: Ratios of the experimental frequency to the unscaled calculated frequency (ν_{exp}/ν_{calc}) are plotted against the unscaled calculated frequency (ν_{calc}) to obtain the Wavenumber-Linear Scaling (WLS) equation.

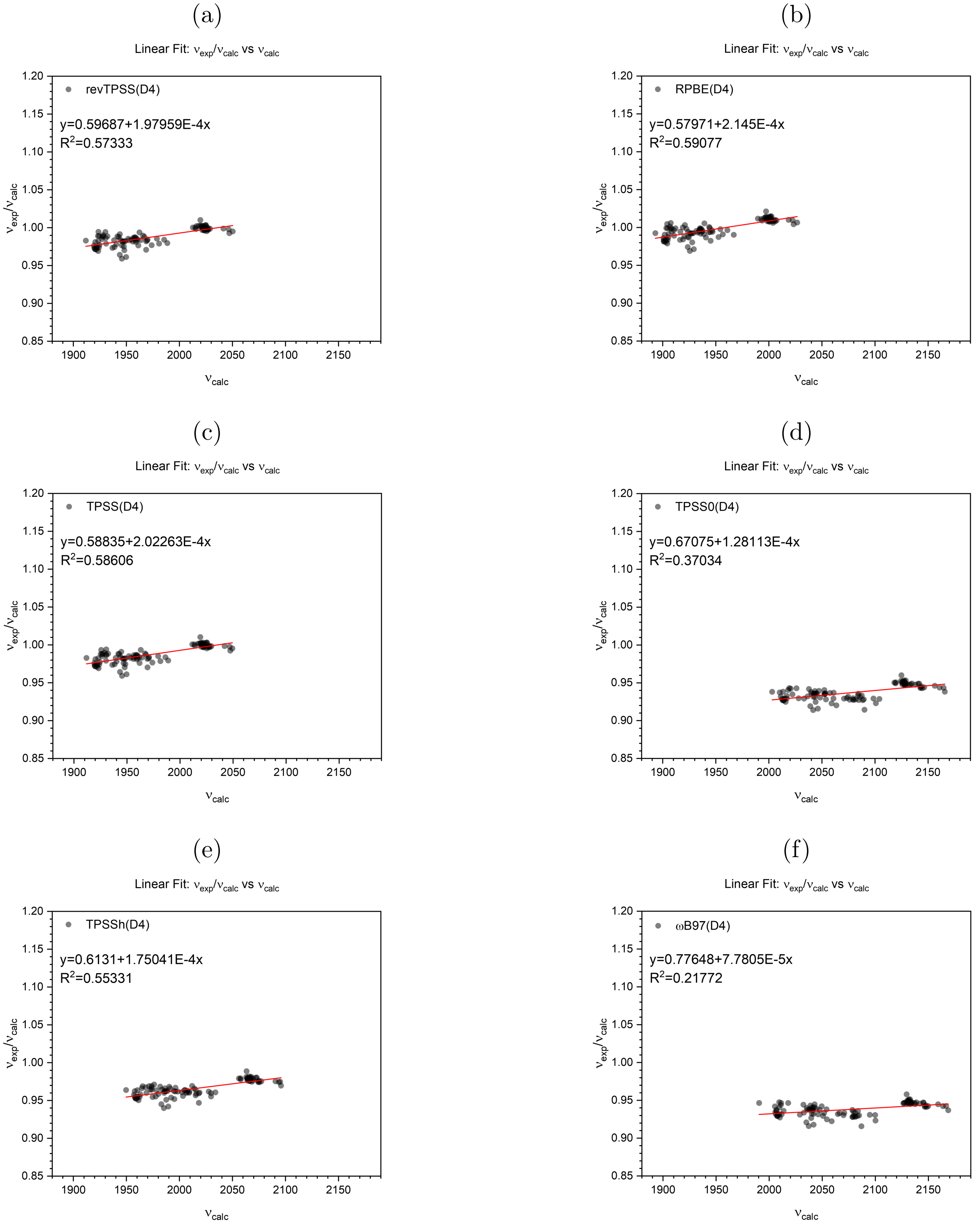

Figure S7: Ratios of the experimental frequency to the unscaled calculated frequency (ν_{exp}/ν_{calc}) are plotted against the unscaled calculated frequency (ν_{calc}) to obtain the Wavenumber-Linear Scaling (WLS) equation.

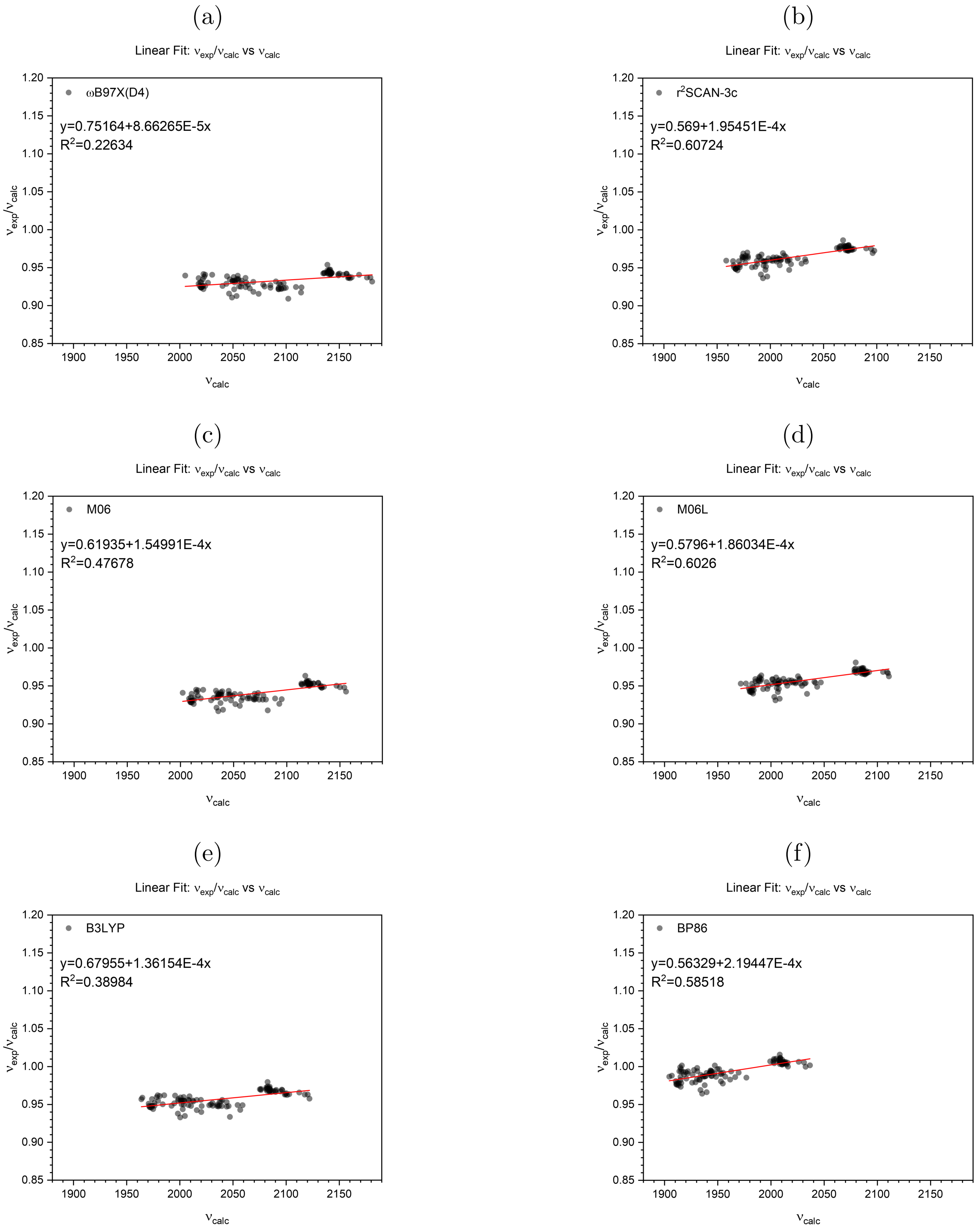

Figure S8: Ratios of the experimental frequency to the unscaled calculated frequency (ν_{exp}/ν_{calc}) are plotted against the unscaled calculated frequency (ν_{calc}) to obtain the Wavenumber-Linear Scaling (WLS) equation.

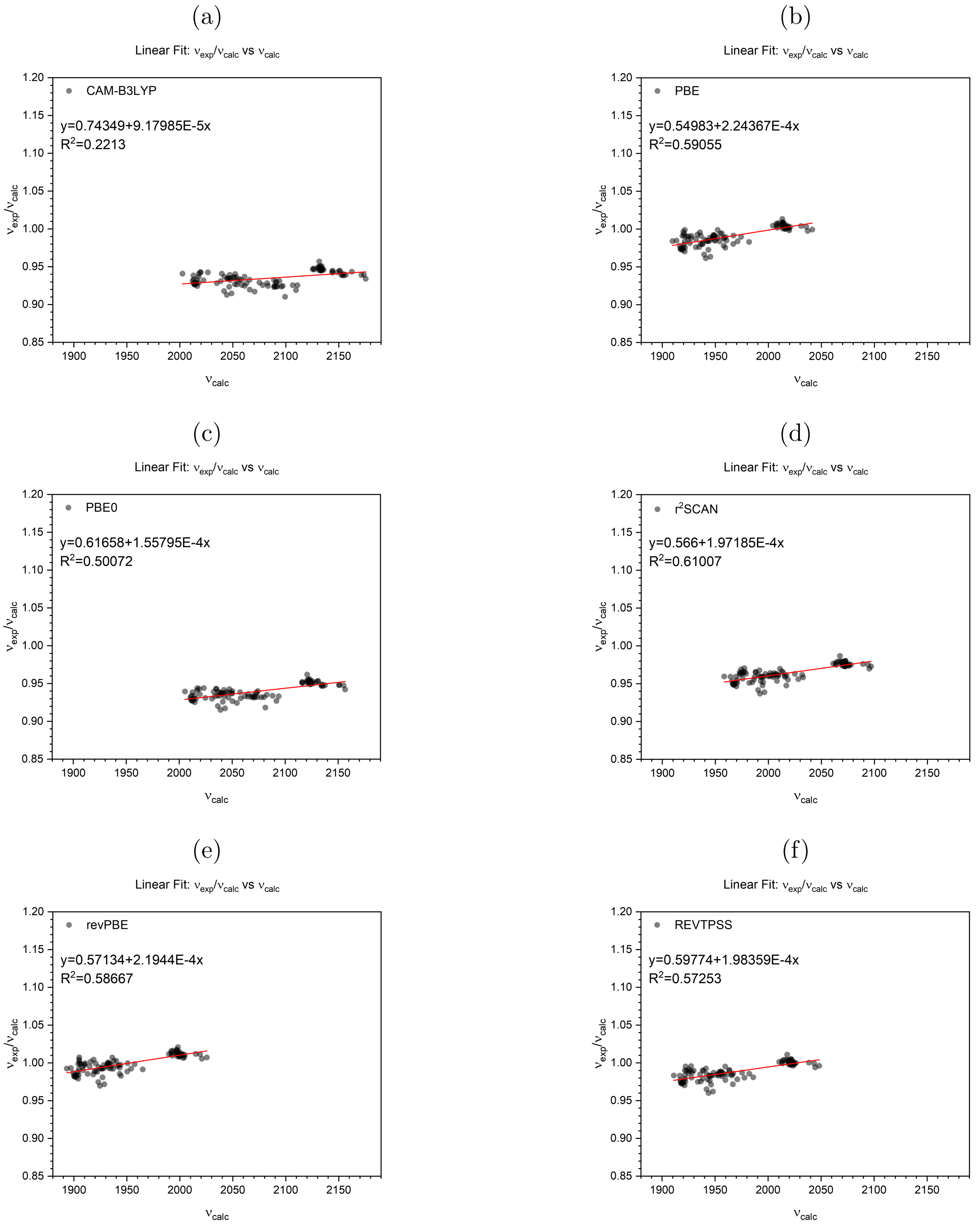

Figure S9: Ratios of the experimental frequency to the unscaled calculated frequency (ν_{exp}/ν_{calc}) are plotted against the unscaled calculated frequency (ν_{calc}) to obtain the Wavenumber-Linear Scaling (WLS) equation.

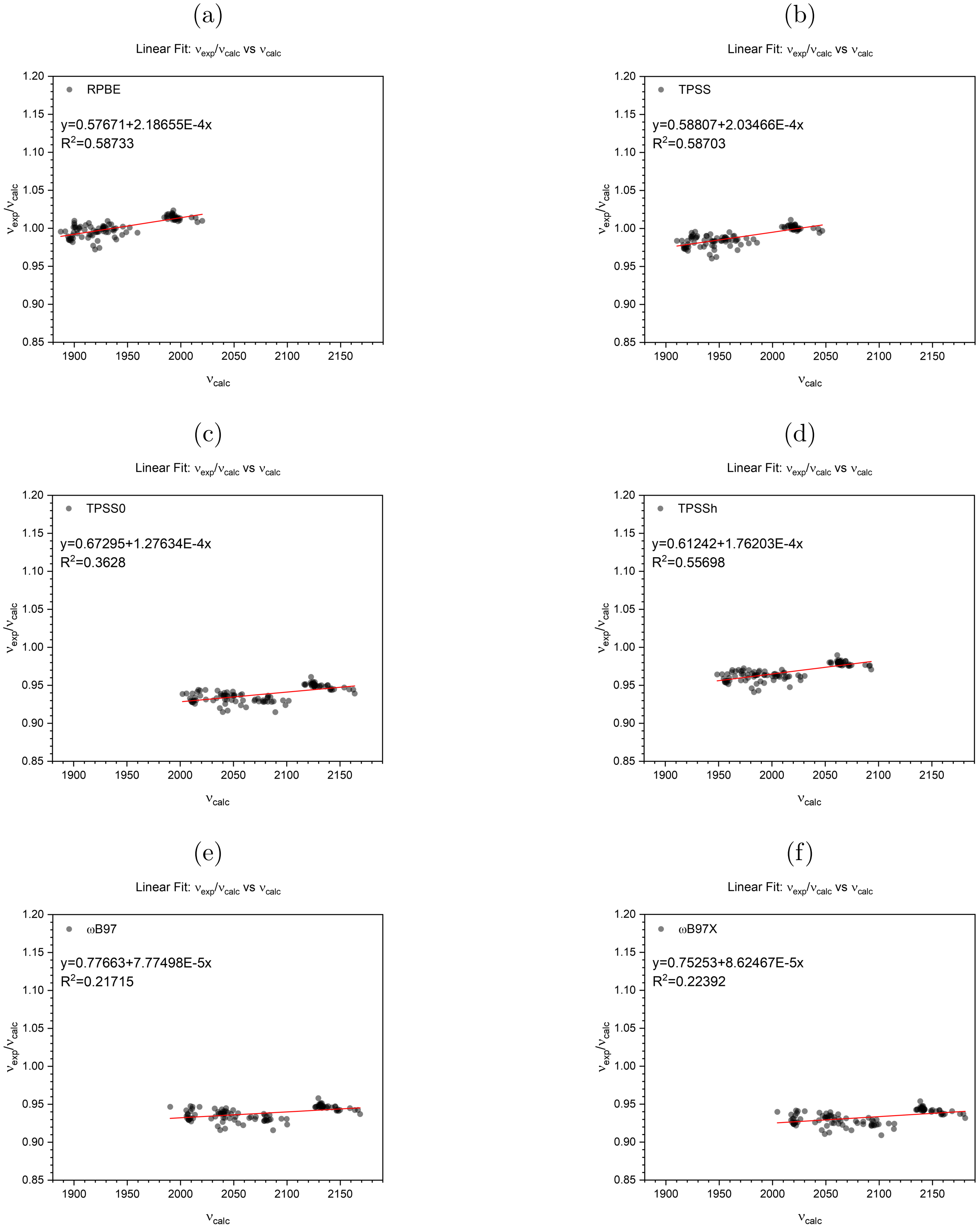

Figure S10: Ratios of the experimental frequency to the unscaled calculated frequency (ν_{exp}/ν_{calc}) are plotted against the unscaled calculated frequency (ν_{calc}) to obtain the Wavenumber-Linear Scaling (WLS) equation.

Table S6: Imaginary vibrational frequencies (cm^{-1}) and their attributions.

Compound	Functional	Value	Attribution
		D4	
**14**	revTPSS	-10.27	rotation of the CF_3 group of the ligand and bending of the entire structure
**15**	revTPSS	-14.91	rotation of the CF_3 group of the ligand and bending of the entire structure
**18**	B3LYP	-7.07	rotation of the CH_3 group of acetonitrile
**18**	PBE0	-6.62	rotation of the CH_3 group of acetonitrile
**18**	r^2SCAN	-12.99	rotation of the CH_3 group of acetonitrile
**18**	revPBE	-6.46	rotation of the CH_3 group of acetonitrile
**18**	revTPSS	-57.49	rotation of the CH_3 group of acetonitrile
**18**	TPSS0	-7.68	rotation of the CH_3 group of acetonitrile
**18**	TPSSh	-9.59	rotation of the CH_3 group of acetonitrile
**18**	TPSS	-12.24	rotation of the CH_3 group of acetonitrile
**18**	ωB97	-3.79	rotation of the CH_3 group of acetonitrile
**18**	ωB97X	-13.30	rotation of the CH_3 group of acetonitrile
**23**	revTPSS	-35.62	rotation of two CH_3 groups of the ligand
**23**	revTPSS	-34.95	rotation of two CH_3 groups of the ligand
**28**	revTPSS	-44.86	rotation of two CH_3 groups of the ligand
**28**	revTPSS	-44.50	rotation of two CH_3 groups of the ligand
**30**	revTPSS	-51.40	rotation of the CH_3 group of the ligand
**30**	revTPSS	-50.29	rotation of the CH_3 group of the ligand
**32**	revTPSS	-49.50	rotation of the CH_3 group of the ligand
**32**	ωB97	-1.67	rotation of the CH_3 group of the ligand
		D3zero	
**15**	M06L	-5.95	rotation of the CF_3 group of the ligand and bending of the entire structure
**15**	M06L	-4.18	bending of the entire structure
**18**	B3LYP	-9.02	rotation of the CH_3 group of acetonitrile
**18**	BP86	-3.96	rotation of the CH_3 group of acetonitrile
**18**	PBE0	-9.90	rotation of the CH_3 group of acetonitrile
**18**	PBE	-3.37	rotation of the CH_3 group of acetonitrile
**18**	revPBE	-4.87	rotation of the CH_3 group of acetonitrile
**18**	RPBE	-9.44	rotation of the CH_3 group of acetonitrile
**18**	TPSS0	-11.67	rotation of the CH_3 group of acetonitrile
**18**	TPSSh	-11.94	rotation of the CH_3 group of acetonitrile
**18**	TPSS	-12.93	rotation of the CH_3 group of acetonitrile
**22**	M06	-5.89	bending of the entire structure
**23**	M06	-62.35	rotation of two CH_3 groups of the ligand
**26**	M06	-11.75	bending of the entire structure

Table S6 – continued from previous page.

Compound	Functional	Value	Attribution
			D3BJ
**18**	B3LYP	-14.13	rotation of the CH_3 group of acetonitrile
**18**	PBE0	-9.81	rotation of the CH_3 group of acetonitrile
**18**	r^{2}SCAN	-12.68	rotation of the CH_3 group of acetonitrile
**18**	RPBE	-2.49	rotation of the CH_3 group of acetonitrile
**18**	TPSS0	-8.03	rotation of the CH_3 group of acetonitrile
**18**	TPSSh	-11.06	rotation of the CH_3 group of acetonitrile
**18**	TPSS	-10.46	rotation of the CH_3 group of acetonitrile
			without dispersion
**14**	revTPSS	-10.46	rotation of the CF_3 group of the ligand and bending of the entire structure
**14**	TPSS	-5.33	rotation of the CF_3 group of the ligand and bending of the entire structure
**15**	M06L	-5.47	rotation of the CF_3 group of the ligand and bending of the entire structure
**15**	M06L	-3.49	bending of the entire structure
**15**	revTPSS	-14.91	rotation of the CF_3 group of the ligand
**18**	PBE0	-7.75	rotation of the CH_3 group of acetonitrile
**18**	r^{2}SCAN	-14.37	rotation of the CH_3 group of acetonitrile
**18**	revTPSS	-57.17	rotation of the CH_3 group of acetonitrile
**18**	TPSS0	-9.43	rotation of the CH_3 group of acetonitrile
**18**	TPSSh	-12.02	rotation of the CH_3 group of acetonitrile
**18**	TPSS	-3.71	rotation of the CH_3 group of acetonitrile
**18**	ωB97	-7.25	rotation of the CH_3 group of acetonitrile
**18**	ωB97X	-7.82	rotation of the CH_3 group of acetonitrile
**19**	M06	-7.69	rotation of the phenyl group and bending of the entire structure
**23**	M06	-98.64	rotation of two CH_3 groups of the ligand
**23**	revTPSS	-35.95	rotation of two CH_3 groups of the ligand
**23**	revTPSS	-35.72	rotation of two CH_3 groups of the ligand
**23**	TPSS	-3.05	rotation of the CH_3 group of the ligand
**26**	M06	-1.30	bending of the entire structure
**28**	revTPSS	-45.52	rotation of the CH_3 group of the ligand
**28**	revTPSS	-45.01	rotation of the CH_3 group of the ligand
**30**	revTPSS	-51.74	rotation of the CH_3 group of the ligand
**30**	revTPSS	-51.35	rotation of the CH_3 group of the ligand
**32**	revTPSS	-54.45	rotation of the CH_3 group of the ligand
**18**	r^{2}SCAN-3c	-15.53	rotation of the CH_3 group of acetonitrile
